# Small Things Matter: The 11.6-kDa TraB Protein is Crucial for Antibiotic Resistance Transfer Among Enterococci

**DOI:** 10.3389/fmolb.2022.867136

**Published:** 2022-04-25

**Authors:** Tamara M.I. Berger, Claudia Michaelis, Ines Probst, Theo Sagmeister, Lukas Petrowitsch, Sandra Puchner, Tea Pavkov-Keller, Bernd Gesslbauer, Elisabeth Grohmann, Walter Keller

**Affiliations:** ^1^ Institute of Molecular Biosciences, Department of Structural Biology, University of Graz, Graz, Austria; ^2^ Faculty of Life Sciences and Technology, Department of Microbiology, Berliner Hochschule für Technik, Berlin, Germany; ^3^ Division of Infectious Diseases, University Medical Center Freiburg, Freiburg, Germany; ^4^ Field of Excellence BioHealth, University of Graz, Graz, Austria; ^5^ BioTechMed-Graz, Graz, Austria; ^6^ Institute of Pharmaceutical Sciences, Division of Pharmaceutical Chemistry, University of Graz, Graz, Austria

**Keywords:** type IV secretion system, *Enterococcus*, plasmid, antibiotic resistance, conjugative transfer, secretion complex

## Abstract

Conjugative transfer is the most important means for spreading antibiotic resistance genes. It is used by Gram-positive and Gram-negative bacteria, and archaea as well. Conjugative transfer is mediated by molecular membrane-spanning nanomachines, so called Type 4 Secretion Systems (T4SS). The T4SS of the broad-host-range inc18-plasmid pIP501 is organized in a single operon encoding 15 putative transfer proteins. pIP501 was originally isolated from a clinical *Streptococcus agalactiae* strain but is mainly found in *Enterococci*. In this study, we demonstrate that the small transmembrane protein TraB is essential for pIP501 transfer. Complementation of a markerless pIP501∆*traB* knockout by *traB* lacking its secretion signal sequence did not fully restore conjugative transfer. Pull-downs with Strep-tagged TraB demonstrated interactions of TraB with the putative mating pair formation proteins, TraF, TraH, TraK, TraM, and with the lytic transglycosylase TraG. As TraB is the only putative mating pair formation complex protein containing a secretion signal sequence, we speculate on its role as T4SS recruitment factor. Moreover, structural features of TraB and TraB orthologs are presented, making an essential role of TraB-like proteins in antibiotic resistance transfer among Firmicutes likely.

## Introduction

Currently, antibiotic resistances are one of the top global threats challenging the world. In recent decades, inappropriate prescription of antimicrobials in health-care settings and overuse along the food chain have led to an increased dissemination of antibiotic resistant (ABR) bacteria (Kim et al., 2021; Lin et al., 2021). Limitations in therapeutical treatment options harden the treatment success of patients with drug-resistant infections caused by hospital-acquired pathogens. The opportunistic Gram-positive (Gram+) pathogens *Enterococcus faecalis* and *Enterococcus faecium* cause the majority of healthcare-associated enterococcal infections (García-Solache and Rice, 2019; Krawczyk et al., 2021; Levitus et al., 2022). Conjugative transfer, a type of horizontal gene transfer, is the major mechanism disseminating antibiotic resistance genes. It is mediated by mobile genetic elements, for example, integrative conjugative elements (ICEs) and conjugative plasmids encoding all the proteins required for their transfer themselves. Even though conjugative elements and their transfer processes are present in both Gram-negative (Gram-) and Gram + bacteria, Gram-conjugation has been studied in more detail (Christie, 2016; Kohler et al., 2018b; Grohmann et al., 2018; Fischer et al., 2020). Profound knowledge on plasmid-mediated conjugation in Gram+ bacteria is highly needed (Alvarez-Martinez and Christie, 2009; Goessweiner-Mohr et al., 2014a; Kohler et al., 2018b; Grohmann et al., 2018; Costa et al., 2021). Membrane-spanning molecular nanomachines like Type IV secretion systems (T4SS) largely contribute to the dissemination of multi-resistant bacteria. They play a primary role in conjugative transfer in Gram- and Gram+ bacteria and are thereby involved in the pathogenesis of bacteria. The main function of T4SSs is the translocation of single-stranded plasmid DNA through the secretion channel, the so-called mating pair formation (MPF) complex, into the recipient.

The plasmid with the broadest known host range among Gram+ conjugative plasmids is the enterococcal plasmid pIP501 which was originally isolated from *Streptococcus agalactiae* (Horodniceanu et al., 1976). It belongs to the incompatibility (inc) group 18 (Palmer et al., 2010). Representatives of the inc18 family encode resistances to the macrolide, lincosamide, and streptogramin (MLS) group of antibiotics. The conjugation machinery of pIP501 is encoded by the ∼15 kb-transfer (*tra*) operon consisting of 15 *tra* genes*, traA* to *traO.* The plasmid is capable of self-transfer and stable replication in virtually all Gram+ bacteria including *Enterococci, Staphylococci, Listeria* or *Streptomyces* and can transfer to Gram- *Escherichia coli* as well (Kurenbach et al., 2003; Kohler et al., 2018b). Seven putative VirB/D4 orthologs of the prototype *Agrobacterium tumefaciens* T4SS have been identified in pIP501 (Grohmann et al., 2003; Goessweiner-Mohr et al., 2014a; Grohmann et al., 2017) thereby representing one of the best-characterized T4SS in Gram+ bacteria (Li and Christie, 2018).

Expression of the 15 pIP501 *tra* genes is regulated by binding of the TraA relaxase to the main conjugation promoter P_*tra*_ (Grohmann et al., 2016). The pIP501 type IV coupling protein (T4CP) consists of two proteins, TraI and TraJ. TraI functions as membrane anchor while TraJ is the canonical coupling factor (Goessweiner-Mohr et al., 2014a). TraG is the VirB1-like lytic transglycosylase of the system. It locally cleaves the peptidoglycan and is involved in the assembly of the MPF proteins (Arends et al., 2013; Kohler et al., 2018b). Eight of the Tra proteins, TraB, TraC, TraF, TraH, TraI, TraK, TraL, and TraM, have been predicted to be involved in the formation of the MPF complex (Kohler et al., 2018b), for example, the two VirB8 orthologs, TraH and TraM, which were postulated to be part of the translocation channel (Kohler et al., 2017; Kohler et al., 2018b). The small cytosolic TraN protein was characterized as conjugation repressor and transcriptional regulator of the *tra* operon. *traN* deletion resulted in enhanced conjugative transfer (Kohler et al., 2018a).

In this study, we focused on the small transmembrane protein TraB. The processed *traB* gene product codes only for 80 amino acids, making it the smallest member of the Tra_pIP501_ protein family. We proved that TraB is essential for pIP501 transfer, present its structural features and discuss its putative role in the T4SS complex.

## Materials and Methods

### Bacterial Strains and Growth Conditions

The bacterial strains and plasmids used in this study are listed in Supplementary Table S1. *Escherichia coli* and *Bacillus megaterium* strains as plasmid hosts were cultured in lysogeny broth (LB) under shaking at 37°C, for *B. megaterium* strains baffled flasks were used. *Enterococcus faecalis* strains were routinely grown in brain heart infusion (BHI) broth at 37°C with shaking. 1% w/v agar was added for preparing solid media. For selection, antibiotics were added as indicated in Supplementary Table S1.

### Construction of a *traB* in-Frame Deletion Mutant in pIP501

A pIP501∆*traB* in-frame deletion mutant was constructed in *E. faecalis* JH2-2 using the previously described markerless allelic exchange strategy (Kristich et al., 2007). Oligonucleotides were purchased from Thermo Fisher and are listed in Supplementary Table S2. Restriction enzymes were purchased from New England Biolabs, as well as T4 DNA ligase and Phusion DNA polymerase. The first recombinant construct for *traB* in-frame deletion was generated by amplifying *traB* upstream and downstream flanking regions (1,007 bp and 1,054 bp, respectively) using *E. faecalis* JH2-2 (pIP501) as template with primers containing PstI/XbaI restriction sites for the upstream fragment and BamHI/EcoRI for the downstream fragment. The fragments were sequentially cloned into pUC18 using the corresponding restriction sites to create pUC18-UPS-DWS-*traB. E. coli* DH5α was transformed with the recombinant DNA, clones were selected on LB agar with 100 μg/ml ampicillin. More than 96% of the *traB* coding region was deleted retaining only two intact N-terminal and C-terminal codons of *traB* to prevent polar effects on downstream *tra* genes. The fused flanking regions were excised from pUC18 by digestion with PstI and EcoRI and inserted into the equally digested suicide plasmid pKA, followed by transformation into *E. coli* EC1000, resulting in pKA-UPS-DWS-*traB*. Clones were selected on BHI agar containing 250 μg/ml erythromycin. The correctness of all plasmid constructs was confirmed by Sanger sequencing (Eurofins Genomics Germany GmbH, Ebersberg, Germany). Electrocompetent *E. faecalis* JH2-2 (pIP501) cells were transformed with pKA-UPS-DWS-*traB* by electroporation with a Bio-Rad GenePulser X-Cell using 0.2 cm gap width cuvettes and settings of 2,000 kV, 200 Ω, and 25 µF. Transformants were selected on BHI agar supplemented with 50 μg/ml fusidic acid, 20 μg/ml chloramphenicol, 20 μg/ml erythromycin, 100 μg/ml gentamicin, coated with 100 µl of a 20 mg/ml 5-bromo-4-chloro-3-indolyl-β-D-galactopyranoside (X-gal) solution. Blue colonies were screened for homologous integration of pKA-UPS-DWS-*traB* into pIP501 by PCR. Colonies with integrated pKA-UPS-DWS-*traB* were grown in BHI medium supplemented with 50 μg/ml fusidic acid and 20 μg/ml chloramphenicol three times to stationary phase and finally until an OD_600_ of 0.4. Serial dilutions were spread on MM9YEG agar plates supplemented with 50 μg/ml fusidic acid, 20 μg/ml chloramphenicol and 10 mM DL-p-chlorophenylalanine, coated with 100 µl 20 mg/ml X-gal solution. White colonies were screened for *traB* deletion by PCR. The pIP501∆*traB* mutant was verified by sequencing of the deletion borders.

### Complementation of *E. faecalis* JH2-2 (pIP501∆*traB*)

For *traB* knockout complementation, the *traB* wild type gene with its ribosomal binding site (RBS) was amplified from pIP501 with primers containing BstYI/SalI restriction sites and inserted into the shuttle vector pEU327 followed by transformation into *E. coli* DH5α cells. Selection was carried out on LB agar with 100 μg/ml spectinomycin. For detection of C-terminally Strep-tag II-coupled TraB proteins and complementation studies a linker, a C-terminal Strep-tag II coding sequence (SA-WSHPQFEK) and a stop codon were inserted into pEU327 by using the site-directed mutagenesis kit (Q5 site-directed mutagenesis kit, New England Biolabs, Frankfurt am Main, Germany) according to the manufacturer’s instructions using the primers pEU327_Mut_C-Strep fw and pEU327_Mut_C-Strep rev. After DpnI treatment the nicked DNA was transformed into *E. coli* DH5α cells, resulting in pEU327 containing the C-terminal Strep-tag (pEU327-Strep). To incorporate the *traB* gene with its native RBS into pEU327-Strep, a Gibson Assembly Kit (New England Biolabs) was applied. The primers used for amplification of pEU327-Strep (GA_pEU327_CStrep fw and GA_pEU327-CStrep rev) and *traB* including its RBS (GA_RBS-traB fw and GA_RBS-traB rev) as well as all other primers used for cloning or sequencing are listed in Supplementary Table S2. The protocol was carried out according to the manufacturer’s instructions, resulting in pEU327-RBS-*traB* with a C-terminal Strep-tag (pEU327-RBS-*traB*-Strep). pEU327-RBS-*traB* was used to delete the *traB*_2-30_ sequence by Q5 site-directed mutagenesis according to the manufacturer’s instructions using the primers Mut_pEUtraB31-110 fw and Mut_pEUtraB31-110 rev, resulting in pEU327-RBS-*traB*_31-110_. For biparental mating assays, *E. faecalis* JH2-2 (pIP501∆*traB*) was electroporated with either pEU327-RBS-*traB,* pEU327-RBS-*traB*-Strep or pEU327-RBS-*traB*_31-110_ as described in *Construction of a traB in-Frame Deletion Mutant in pIP501* Selection for *E. faecalis* JH2-2 (pIP501∆*traB;* pEU327-RBS-*traB*), *E. faecalis* JH2-2 (pIP501∆*traB;* pEU327-RBS-*traB*-Strep) and *E. faecalis* JH2-2 (pIP501∆*traB;* pEU327-RBS-*traB*_31-110_) was conducted on BHI agar with 20 μg/ml chloramphenicol, 20 μg/ml erythromycin and 500 μg/ml spectinomycin. Transformants were confirmed by PCR analysis.

### Biparental Mating Assay

Biparental mating assays were performed with isogenic *E. faecalis* (pIP501), *E. faecalis* (pIP501∆*traB*), *E. faecalis* (pIP501∆*traB;* pEU327-RBS-*traB*), *E. faecalis* JH2-2 (pIP501∆*traB*, pEU327-RBS-*traB-*Strep) and *E. faecalis* JH2-2 (pIP501∆*traB*, pEU327-RBS-*traB*_31-110_) as donor strains and *E. faecalis* OG1X as plasmid-free recipient as described in Fercher et al. (2016). Transconjugants were selected on BHI agar supplemented with 1.5 mg/ml streptomycin and 20 μg/ml erythromycin. All mating assays were performed in quadruplicates. Transfer rates (number of transconjugants per recipient cell) are given with standard deviation. Significance and *p*-values were calculated with the Welch’s *t* test. Significance is indicated by asterisks: ***p < 0.0003, **p < 0.0038.

### Cloning of *traB*

Based on the secondary structure prediction of TraB a truncation variant of *traB* was generated, lacking the secretion signal sequence. It was denominated TraB_31-110_ referring to the amino acids present in the truncated protein. The 243-bp *traB*_31-110_ PCR product was cut with BamHI and HindIII (New England Biolabs) and ligated into the expression plasmid pQTEV cut with the same enzymes. pQTEV contains a tobacco etch virus (TEV) protease-cleavable His-tag. The recombinant plasmid was transformed into *E. coli* BL21-CodonPlus (DE3)-RIL and BL21 star (DE3). The correctness of the *traB*_31-110_ sequence was verified *via* Sanger sequencing by Microsynth AG (Balgach, Switzerland). All primers used for cloning and sequencing are listed in Supplementary Table S2.

### TraB_31-110_ Expression and Test Purification

TraB_31-110_ was expressed in BL21 star (DE3) and *E. coli* BL21 CodonPlus (DE3)-RIL cells in LB medium supplemented with ampicillin (100 μg/ml) and in addition with chloramphenicol (32 μg/ml) for *E. coli* BL21 CodonPlus (DE3)-RIL under shaking with 180 rpm. At an OD_620_ of ∼0.7 TraB_31-110_ expression was induced by adding 0.5 mM IPTG. Incubation continued for 16 h at 30°C under shaking. Cells expressing TraB_31-110_ were harvested by centrifugation at 9,000 × g for 40 min. The pellet was resuspended in lysis buffer (50 mM Tris/HCl, 150 mM NaCl, 20 mM imidazole, 2% Tween-20, pH 8.0, filtered using a MF-Millipore™ 0.45 µm MCE membrane (Merck, Darmstadt, Germany) containing protease inhibitor (Pierce protease Inhibitor Tablets, Thermo Fisher Scientific, Waltham Massachusetts, United States). After homogenization the cells were disrupted by sonication (50% duty cycle, 60% intensity, Sonopuls HD 2070 ultrasonic probe) on ice four times for 16 min each with 8 min breaks in between. The disrupted cells were centrifuged in a Beckman Coulter Avanti J-26 XP centrifuge (Brea, California, United States) using a JA 25.50 rotor at 38,000 × g for 40 min. The supernatant was filtered through a PVDF Rotilabo^®^ syringe filter with a pore size of 0.45 µm (Carl Roth GmbH + Co. KG, Karlsruhe, Germany) and loaded onto a 5-ml HisTrap™ HP column (Cytiva, Marlborough, Massachusetts, United States) and purified by an ÄKTA FPLC system. Test purification was monitored on a 12% SDS polyacrylamide gel using Lämmli standard buffer conditions (Laemmli, 1970).

### TraB_31-110_ Purification

All purification steps were performed on an ÄKTA FPLC system. Elution fractions of the nickel-based immobilized metal affinity chromatography (Ni-IMAC) were pooled and applied to dialysis using a dialysis tube (Spectra/Por^®^ 3, Repligen, Waltheim, Massachusetts, United States) with a molecular weight (MW) cutoff of 3.5 kDa. TraB_31-110_ was cleaved by TEV protease and dialyzed against 50 mM Tris/HCl, 150 mM NaCl and 2 mM DTT for 2 days. Cleaved TraB_31-110_ was separated from uncleaved TraB_31-110_ and TEV protease by performing a reverse Ni-IMAC using a 5-ml HisTrap™ HP column at a flow rate of 3 ml/min. As last purification step, a size exclusion chromatography (SEC) using a Superdex 200 increase 10/300 (Cytiva) column at a flow rate of 0.3 ml/min was performed.

### Construction of Plasmids for Expression of pIP501 *tra* Operon Proteins in *B. megaterium*

Polycistronic expression of *traB-traO*_pIP501_ in *B. megaterium* MS941 was carried out with the *B. megaterium*/*E. coli* shuttle vector pMGBm19 containing *traB* to *traO. traB* with a C-terminal Strep-tag II and a ribosomal binding site was inserted into the *B. megaterium*/*E. coli* shuttle vector pRBBm59 to allow purification of TraB and its interaction partners *via* affinity chromatography.

*traB* to *traO*_pIP501_ was amplified using Q5 polymerase and primers pMGBm19-traBO_fw and pMGBm19-traBO_rev. The BamHI/SacI-digested insert was ligated into pMGBm19 cut with the same enzymes and transformed into One Shot™ TOP10 Chemically Competent *E. coli* cells, resulting in pMGBm19-RBS-*traB*-*traO*. Gibson Assembly (Gibson Assembly Kit, New England Biolabs) was used to insert RBS-*traB*-Strep into pRBBm59. pEU327-RBS-*traB*-Strep served as template for amplification of the insert. Prior to Gibson Assembly, *traB*-Strep was amplified with an added stop codon, an artificial RBS (AAAGGGGGAAA) and a BamHI restriction site as spacer on the 5’ end using primers traB-pRBBm59 fw and traB-pRBBm59 rev, resulting in a 389 bp fragment. The amplicon was used as a template to produce the Gibson Assembly insert using the primers GA_traB CStrep prBB fw and GA_traB CStrep prBB rev. The backbone of pRBBm59 was amplified by using GA_pRBBm59 fw and GA_pRBBm59 rev primers and Q5 polymerase, generating a 5,750 bp fragment. The resulting construct was denominated pRBBm59-RBS-*traB*-Strep. The correctness of all plasmids generated was confirmed by Sanger sequencing. All primers used for cloning and sequencing are listed in Supplementary Table S2.

*B. megaterium* MS941 was co-transformed with pMBGm19-RBS-*traB*-*traO*, containing a xylose-inducible promoter and pRBBm59-RBS-*traB*-Strep, containing a sucrose-inducible promoter using a protoplast transformation protocol for *B. megaterium* (Biedendieck et al., 2011). Colonies were selected on LB agar containing 35 μg/ml chloramphenicol and 10 μg/ml tetracycline and were screened for the presence of the plasmids by colony PCR.

### Expression and Purification of Strep-Tagged TraB and its Interaction Partners from *B. megaterium* MS941 (pMGBm19-RBS-*traB-traO*; pRBBm59-RBS-*traB*-Strep) Lysate

Overnight cultures of *B. megaterium* MS941 (pMGBm19-RBS-*traB*-*traO*; pRBBm59-RBS-*traB*-Strep) were grown in baffled flasks in LB medium supplemented with chloramphenicol (35 μg/ml) and tetracycline (10 μg/ml) at 37°C and 250 rpm. Expression cultures (1 l in 5 l baffled flasks) were inoculated to an OD_600_ of 0.05 in the same medium and incubated until an OD_600_ of 0.3–0.4 at 37°C. Expression of TraB-TraO and Strep-tagged TraB was induced with 0.5% (w/v) xylose and sucrose, respectively, followed by incubation for 4 h. Cells were harvested at 9,000 × g at 4°C, lysed in 70 ml TraB-TraO/TraB-Strep binding buffer (50 mM Hepes, 300 mM NaCl, 1 mM EDTA, pH 7.5, 0.5% Nonidet^®^ P 40 Substitute (NP-40) Proteomics Grade (AMRESCO, VWR Life Science) filtered through a MF-Millipore 0.45 µm MCE membrane (Merck, Darmstadt, Germany) supplemented with additional NP-40 up to 2% and protease inhibitor (Pierce protease Inhibitor Tablets, Thermo Fisher Scientific, Waltham Massachusetts, United States) using the SONOPULS HD 2070 ultrasonic homogenizer (BANDELIN electronic GmbH & Co. KG, Berlin, Germany) on ice for 60 min at 60% intensity and 50% duty cycle. After 60 min centrifugation at 4°C and 38,000 × g, the supernatant was passed through a 0.45 μm PVDF syringe filter (Merck Chemicals and Life Science GmbH, Vienna, Austria) prior to loading onto a 5-ml StrepTrapTM HP column pre-equilibrated with TraB-TraO/TraB-Strep binding buffer at 4°C using an ÄKTA pure 25 L chromatography system. The protein was eluted in a 60% gradient of TraB-TraO/TraB-Strep elution buffer (50 mM Hepes, 300 mM NaCl, 1 mM EDTA, 2.5 mM desthiobiotin). 2 ml fractions were collected. Peak fractions were loaded onto a 12% SDS polyacrylamide gel and subjected to immunoblotting using polyclonal anti-Tra antibodies directed against TraF, TraG, TraH, TraK, and TraM (Biogenes, Berlin, Germany) at a 1:10,000 dilution to probe for coeluted Tra proteins. As secondary antibody a horseradish peroxidase conjugated antibody against rabbit IgG (Promega GmbH, Mannheim, Germany) was used at a dilution of 1:10,000.

### CD Measurements

CD measurements were performed on a J-1500 Circular Dichroism Spectrophotometer (JASCO Corporation, Tokyo, Japan). Far-UV CD spectra were recorded at 20°C from 260 to 180 nm with a data pitch of 0.2 nm and a bandwidth of 2 nm applying a scanning speed of 100 nm/min. Each spectrum was recorded as an average of 20 scans. The resulting spectra were baseline-corrected by subtracting the signal of the buffer. TraB_31-110_ was prepared with a concentration of 0.15 mg/ml in 50 mM Tris/HCl, 150 mM NaCl, pH 8.0, 0.05% DDM. The CD signal was converted to mean residue ellipticity [θ] and secondary structure was determined with dichroweb using the CDSSTR algorithm (Compton and Johnson, 1986; Miles et al., 2022).

### *In Vitro* Cross-Linking of TraB_31-110_

50 µl samples of 10 µg TraB_31-110_ in SEC buffer containing 0/0.001/0.01/0.05/0.1% glutaraldehyde in 50 mM Bicine, 150 mM NaCl, 1 mM DTT were incubated for 20 min at room temperature. Cross-linking was stopped by adding glycine to a final concentration of 140 mM followed by 5 min incubation at room temperature. 400 µl of −20°C-cold acetone was added and the samples were precipitated at −20°C for 2 h, followed by centrifugation at 16,100 × g for 15 min. Pellets were resuspended in 10 µl distilled water and 10 µl SDS-PAGE loading buffer. 15 µl samples were loaded onto a 12% SDS polyacrylamide gel. Unstained protein MW marker (Thermo Fisher Scientific) was used to assess the size of the cross-linked oligomers.

### Mass Spectrometry of Putative TraB_31-110_ Bands

Putative TraB_31-110_ bands were excised from a silver-stained 12% SDS polyacrylamide gel and subjected to in-gel reduction, alkylation and trypsinization as described previously (Gesslbauer et al., 2018). Extracted peptides were purified and concentrated using C18 spin columns (Pierce™ C18 Spin Columns; Thermo Fisher Scientific) according to the manufacturer’s protocol.

The concentrated purified peptides were applied to Liquid Chromatography (LC) through an Ultimate 3,000 RSLCnano System (Thermo Fisher Scientific). Peptides were separated with a flow rate of 300 nl/min on a C18 Aurora UHPLC column (25 cm × 75 µm ID, 1.6 µm) with CaptiveSpray Insert (IonOptics). The mobile phases were (A) 0.1% (v/v) formic acid in water and (B) 0.1% (v/v) formic acid in acetonitrile. The HPLC gradient for separation was 2% B for 6 min, 2–25% B for 90 min, 25–40% B for 10 min, 40–80% B for 10 min, 80% B for 10 min and 2% B for 12 min. The LC system was coupled online to a timsTOF Pro mass spectrometer (Bruker Corporation, Billerica, Massachusetts, United States) with CaptiveSpray nano-electrospray ion source (Bruker). The mass spectrometer was operated in PASEF mode. TIMS, PASEF and calibration settings were applied as described by Meier et al. (2018).

Mass spectrometry raw files were processed with MaxQuant version 1.6.15.0. MS/MS spectra were searched against the UniProt *E. coli* (K12) reference proteome databank (Proteome ID: UP000000625) (Cox and Mann, 2008). The TraB_31-110_ sequence was manually included in the *E. coli* FASTA file. Search parameters were set as follows: trypsin was set as enzyme; a maximum of two missed cleavages was allowed; the minimum peptide sequence length was seven amino acids, and the maximum peptide mass was 4,600 Da. Carbamidomethylation of cysteine was set as a fixed modification; oxidation of methionine and acetylation of protein amino-termini were set as a variable modification. All other MaxQuant search parameters were equivalent to those described by Meier et al. (2018).

### Structural Characterization of TraB

The following online services were used to search for transmembrane motifs in the TraB sequence and potential orthologous proteins: PSIPRED 4.0 (Buchan and Jones, 2019; Moffat and Jones, 2021), MEMPACK (Nugent et al., 2011), DomPred (Bryson et al., 2007), MEMSAT-SVM (Nugent and Jones, 2009). PSIPRED was used to predict the secondary structure content of TraB and orthologous proteins. After identifying seven TraB orthologs by an iterative PSI-BLAST using the NCBI database, a multiple sequence alignment with T-Coffee was performed using the PSI/TM-Coffee function for membrane proteins (Notredame et al., 2000; Chang et al., 2012). The multiple sequence alignment and the structural categorization were evaluated with the implemented TCS tool (Chang et al., 2014; Chang et al., 2015). General features of the recombinant TraB_31-110_ constructs were assessed with ProtParam (Gasteiger et al., 2005). RoseTTAFold was used for 3D structure prediction of TraB and its putative orthologs (Baek et al., 2021). *PyMOL* Molecular Grahics System, Version 2.5 was used for the presentation of the theoretical structures and their alignment using the method “cealign” (Shindyalov and Bourne, 1998).

### Subcellular Localization of TraB in *E. faecalis*

Subcellular fractionation of *E. faecalis* JH2-2 (pIP501), *E. faecalis* JH2-2 (pIP501∆*traB*), and *E. faecalis* JH2-2 (pIP501∆*traB*, pEU327-RBS-*traB*-Strep) was performed as described in Goessweiner-Mohr et al. (2013) with some modifications. Cultures were diluted to an OD_600_ of 0.05 in BHI medium containing 20 μg/ml chloramphenicol, for the pEU327 derivative, additionally supplemented by 500 μg/ml spectinomycin and incubated at 37°C until an OD_600_ of 0.5 was reached. The culture was kept on ice for 15 min and centrifuged for 10 min at 4°C at 6,000 × g. The pellet was washed twice with 10 ml 50 mM KH_2_PO_4_/K_2_HPO_4_ buffer (50 mM, pH 7) and resuspended in 1 ml lysis buffer (50 mM KH_2_PO_4_/K_2_HPO_4_, pH 7, 1 mM EDTA, 1 mM MgCl_2_, 100 μg/ml RNAse, 1 U/µl DNAse, 1:1000 diluted protease inhibitor [stock solution: 1 mg/ml pepstatin, 2 mg/ml antipain, 20 mg/ml leupeptin], 1 mg/ml lysozyme). Unlysed material was removed by centrifugation at 1,000 × g for 2 min at 4°C. The supernatant was centrifuged at 16,000 × g for 20 min at 4°C. The resulting pellet corresponding to the cell wall fraction was resuspended in 100 µl potassium phosphate buffer (50 mM KH_2_PO_4_/K_2_HPO_4_, pH 7). The supernatant was applied to ultracentrifugation at 163,000 × g for 2 h at 4°C. The resulting pellet representing the membrane fraction was resuspended in 100 µl potassium phosphate buffer (50 mM KH_2_PO_4_/K_2_HPO_4_, pH 7) containing 1% Triton X-100. The supernatant containing the cytoplasmic fraction was reduced to a final volume of 100 µl by vacuum centrifugation in a centrivap concentrator (LABCONCO, Kansas City, Missouri, United States). TraB was probed in the cell wall, cell membrane, and cytoplasmic fraction by western blotting using a polyclonal antibody directed against TraB_31-110_ (ProteoGenix SAS, Schiltigheim, France) in a dilution of 1:8,000. The same protocol was used as described in (*Expression and Purification of Strep-Tagged TraB and its Interaction Partners from *B. megaterium* MS941 (pMGBm19-RBS-* traB-traO*; pRBBm59-RBS-*traB*-Strep) Lysate*), with the difference that no detergent was used processing the membrane.

## Results

### TraB is Essential for pIP501 Transfer

**FIGURE 1:**
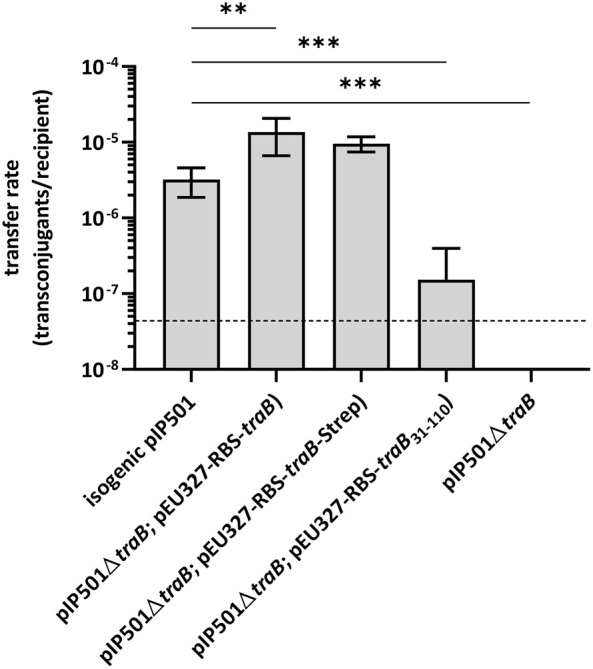
Results of biparental mating assays of pIP501 and derivatives. *E. faecalis* JH2-2 isogenic wild type (pIP501), mutant strain *E. faecalis* JH2-2 (pIP501∆*traB*) and complementation strain where *traB, traB* with a C-terminal Strep-tag or *traB*_31-110_ are supplied *in trans* (*E. faecalis* JH2-2 (pIP501∆*traB*; pEU327-RBS-*traB*), *E. faecalis* JH2-2 (pIP501∆*traB*; pEU327-RBS-*traB*-Strep) or *E. faecalis* JH2-2 (pIP501∆*traB*; pEU327-RBS-*traB*_31-110_) were applied as donors and *E. faecalis* OG1X as recipient. Transfer frequencies are presented as the number of transconjugants per recipient cell. n = 4. Mean values are depicted with the standard deviation. ***p < 0.0003, **p < 0.0038 as determined by Welch’s *t* test. The dashed line depicts the detection limit of the assay (4.4 × 10^−8^ transconjugants per recipient).

To study the role of the transmembrane protein TraB in pIP501 conjugative transfer, biparental mating assays were conducted with the *E. faecalis* JH2-2 (pIP501∆*traB*) deletion mutant as donor and *E. faecalis* OG1X as recipient. The transfer frequency of the deletion mutant was below the detection limit of the assay (<4.4 × 10^−8^ transconjugants per recipient). As *traB* deletion resulted in a total loss of plasmid transfer, TraB proved to be essential for pIP501-mediated conjugative transfer (Figure 1).

Complementation of the *traB* knockout by supplying the wild type *traB* gene *in trans* showed full recovery of transfer with transfer frequencies close to wild type level thus excluding polar effects of the deletion on downstream *tra* genes. Similar transfer rates were observed, when supplying the wild type *traB* gene with a C-terminal Strep-tag (*traB*-Strep) *in trans*. Thus, the Strep-tag did not impact the functionality of TraB. Complementation of the deletion mutant by supplying a truncated *traB*_31-110_ variant lacking the N-terminal secretion signal sequence *in trans* resulted in a significantly diminished transfer rate in comparison to the wild type pointing to a crucial role of the signal sequence as correct localization of TraB is substantial in the transfer process.

### TraB is an α-Helical Transmembrane Protein

**FIGURE 2:**
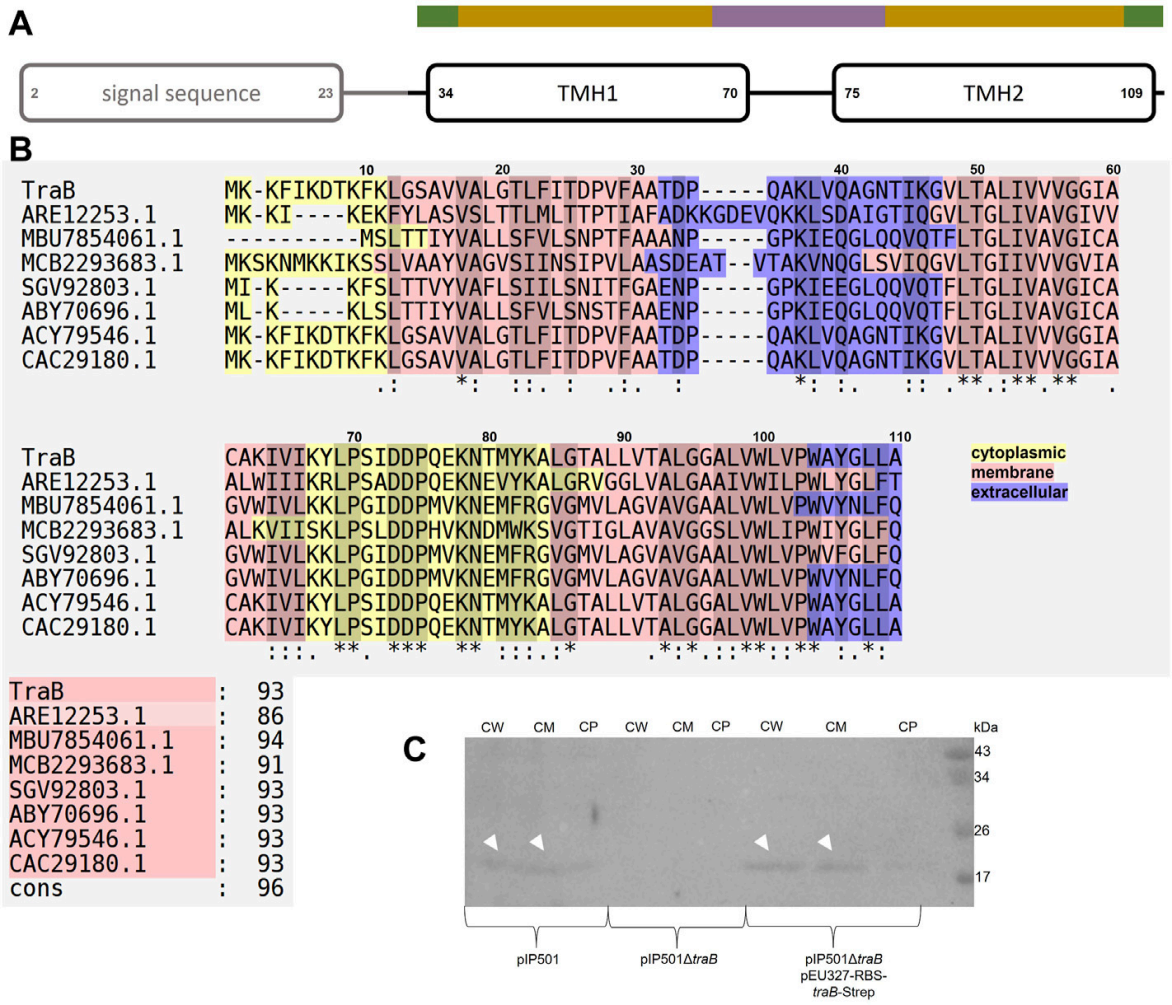
**(A)** Domain composition of TraB. The first predicted helix is part of the secretion signal sequence (predicted with SignalP5.0) including a cleavage site between alanine 30 and alanine 31. Secondary structure elements were calculated with PSIPRED. After a short coil structure, the first transmembrane helix (TMH1) spans from proline 34 to proline 70 followed by a short loop region leading to the second transmembrane helix (TMH2) starting at proline 75 and ending at leucine 109. MEMPACK and MEMSAT-SVM were used to determine the orientation of TraB in the membrane as represented by the color code above the TMHs. Green parts are predicted to be extracellular, golden parts within the membrane and the lavender part intracellular. **(B)** Multiple sequence alignments using PSI/TM-Coffee for membrane proteins from the T-coffee server of TraB and its orthologs from other putative conjugative systems. Orthologs were identified by an iterative PSI-BLAST using the NCBI databank. Only proteins originating from a conjugative context were included in the multiple sequence alignments. The color scheme shows the orientation of the proteins mapped on to their sequence. The panel to the right shows the evaluation scores from the TCS evaluation. Asterisks “*” show conserved amino acids in all sequences. Colons “:” indicate amino acids of high similarities. Dots “.” indicate partial similarities. In addition, identical and highly similar amino acids are highlighted with grey shading of the respective alignment boxes. The numbers above the aligned sequences are referring to the amino acid position of TraB_pIP501_ **(C)** Western Blot of the cell fractionation of *E. faecalis* JH2-2 harboring different plasmids using a specific antibody against TraB_pIP501_. The plasmids are given below the membrane. The fractions are labeled as: CW: cell wall; CM: cell membrane; CP: cytoplasm. TraB (white arrow heads) shows an unusual migration behavior with an apparent MW of 20 kDa on the SDS-PAGE, which is commonly observed for membrane proteins and may be explained by the atypical SDS binding (Rath et al., 2009).

Combining the secondary structure prediction of PSIPRED and the analysis with MEMPACK, MEMSAT-SVM and SignalP5.0 a scheme of the TraB domain composition was built (Nugent and Jones, 2009; Nugent et al., 2011; Almagro Armenteros et al., 2019; Buchan and Jones, 2019; Moffat and Jones, 2021) (Figure 2). Although TraB is predicted to contain three α-helices, the mature TraB_31-110_ only contains two membrane spanning helices as the first helix spanning from lysine 2 to phenylalanine 23 belongs to a secretion signal sequence, which is cleaved off between alanine 30 and alanine 31.

The first transmembrane helix (TMH1) starts at proline 34 and stops at proline 70. It is predicted to enter the membrane at asparagine 43 and leaves the membrane at isoleucine 64. Lysine 67 might interact with the phospholipid headgroup and tyrosine 68 as a membrane anchor. The loop connecting TMH1 and TMH2 is facing into the cytosol. TMH2 starts with proline 75, is predicted to enter the cell membrane at leucine 85 and to leave it at tyrosine 106. Tryptophane 104 probably serves as membrane anchor. The possible function of some particular amino acids will be discussed in more detail in the following chapters.

### TraB_pIP501_ Localizes in the Cell Envelope

Subcellular localization by cell fractionation revealed that TraB_pIP501_ is found in the cell wall fraction and in the cell membrane fraction. This has been shown for *E. faecalis* JH2-2 (pIP501) as well as for the complementation strain *E. faecalis* JH2-2 (pIP501∆*traB*, pEU327-RBS-*traB*-Strep). The *traB* deletion strain *E. faecalis* JH2-2 (pIP501∆*traB*) served as negative control. The Western Blot is shown in Figure 2, panel C.

### TraB Orthologs Were Found on Type IV Secretion Systems from Diverse Firmicutes

We used the NCBI database to search for TraB_pIP501_ orthologs. The results are shown in Table 1. Interestingly, TraB orthologs were detected on numerous bacterial strains belonging to the phylum Firmicutes, including nosocomial pathogens such as MRSA, *S. aureus*, *E. faecalis* as well as biotechnologically relevant bacteria such as *Lactococcus lactis* and environmental species like *Clostridium algoriphilum*. Their identities range from 42 to 100% identity on amino acid level. All *traB* orthologs were found in gene clusters encoding a putative T4SS, several of them on conjugative plasmids, namely on the broad-host-range inc18 plasmids, pRE25 and pAMβ1 (TraB orthologs with 100% identity on amino acid level; the other Tra orthologs show identities in the range of 74–100%) from *E. faecalis*, on the pSK41/pGO1-family plasmid pV030-8 from *S. aureus* and on the pNP40-like plasmid pUC11B from *Lactococcus lactis* (Ortiz Charneco et al., 2021). All of them are small proteins containing 101 to 114 amino acids in their unprocessed form. Their mature form is even more similar regarding their size ranging from 80 to 83 amino acids.

**TABLE 1:** TraB_pIP501_ and its orthologs found with PSI-BLAST using the NCBI database.

origin	Accession Number	Length (aa)	Query Coverage [%]	identity [%]	Gene Product
pIP501	AAA99467.1	110	—	—	TraB_pIP501_
pUC11B	ARE12253.1	111	70	57.14	mating channel formation protein
*Staphylococcus aureus* AF2236	MBU7854061.1	101	90	45.45	CagC family T4SS protein
*Clostridium algoriphilum* DSM 16153	MCB2293683.1	114	90	45.0	CagC family T4SS protein
*Staphylococcus aureus* 3688STDY6124879	SGV92803.1	105	93	41.75	TraB
pV030-8	ABY70696.1	105	92	44.12	TrsB
pAMβ1	ACY79546.1	110	100	100	TrsB
pRE25	CAC29180.1	110	100	100	TrsB

If encountered on a plasmid, the plasmid name is given, if location of the gene (genome or plasmid) is not specified, the bacterial strain is given. The table includes the length of the orthologs in number of amino acids (aa), the name/putative function of the protein. All orthologs are part of a putative type IV secretion system gene cluster.

Figure 2, panel B shows a multiple sequence alignment generated by PSI/TM-Coffee of TraB_pIP501_ and its orthologs presented in Table 1. The accession numbers of the respective proteins are given. Table 1 gives both, the accession number, and the name of the respective gene product. The multiple sequence alignment suggests which parts of the proteins are inserted into the membrane. All TraB orthologs show the same pattern according to their insertion into the membrane, with the N- and C-terminal region pointing to the extracellular space and a loop region connecting two transmembrane helices facing into the cytoplasm (note: The amino acids 2–23 of TraB comprise the signal sequence). The evaluation with the in the T-Coffee package implemented calculation of the transitive consistency score (TCS) shows a high score for every protein sequence, rendering the prediction and the multiple sequence alignment reliable (Chang et al., 2014; Chang et al., 2015). The mating channel formation protein from pUC11B shows the lowest overall identity with TraB_pIP501_, which is also reflected by the TCS evaluation. The largest sequence differences are located at the N-terminal part containing the secretion signal sequences. The best-conserved region is the part facing into the cytoplasm, although it has a difference of two amino acids in length. The second transmembrane helix of TraB from *Staphylococcus aureus* 3688STDY6124879 and the CagC family T4SS protein of *Clostridium algoriphilum* DSM 16153 seem to be longer than the second transmembrane helix of the other ones.

### The Theoretical Structures of TraB and Its Orthologs Show High Similarity

**FIGURE 3:**
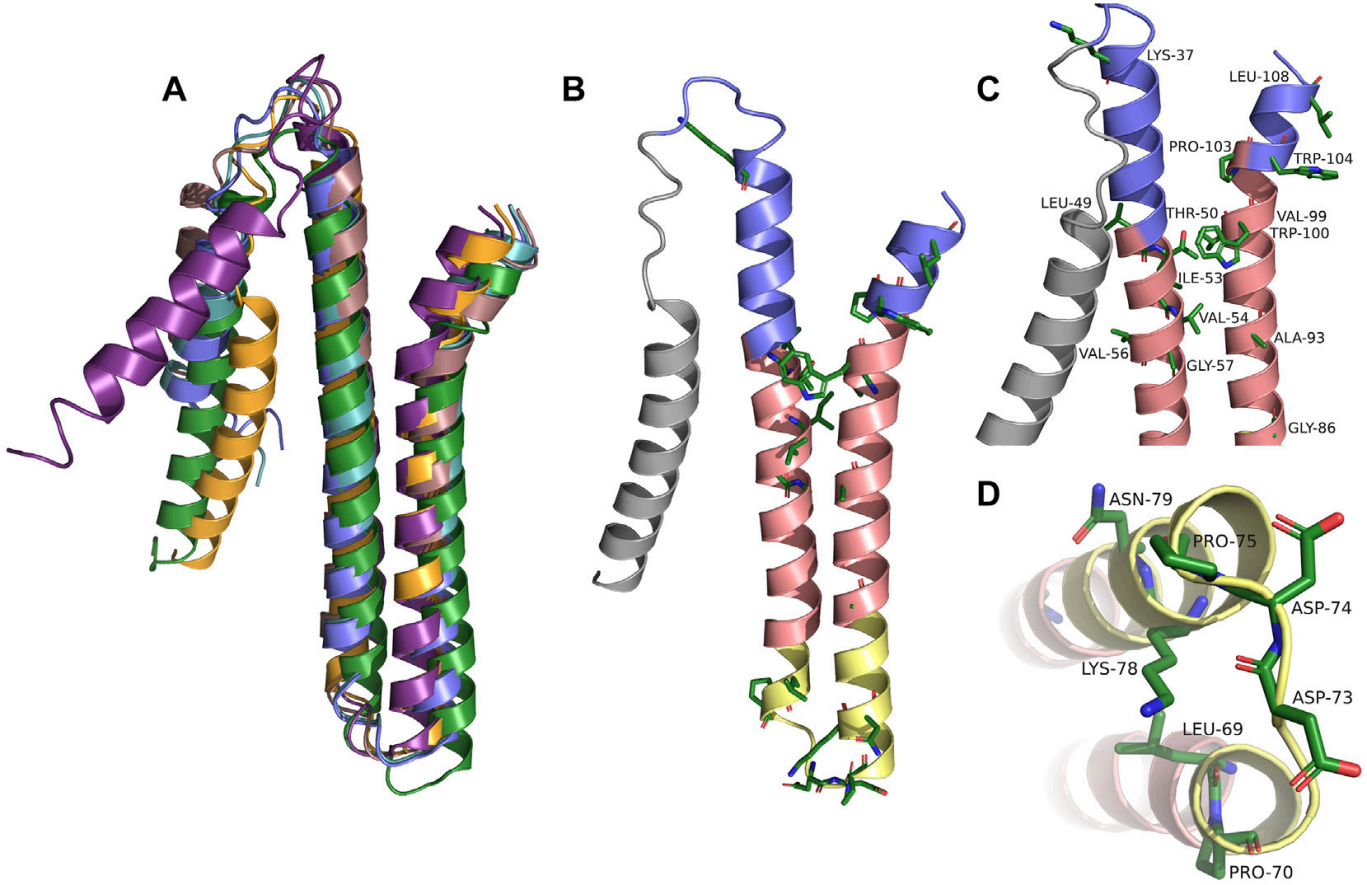
**(A)** Alignment of TraB_pIP501_ and its orthologs using the theoretical structures from Robetta. TrsB_pAMβ1_ and TrsB_pRE25_ were not included, because both are 100% identical to TraB_pIP501_. TraB_pIP501_ is shown in orange; the mating channel formation protein from pUCB11B is shown in purple; the CagC family protein from *Staphylococcus aureus* AF2236 is shown in rose; the CagC family protein from *Clostridium algoriphilum* DSM 16153 is depicted in green; TraB from *Staphylococcus aureus* 3688STDY6124879 is shown in slate; TrsB_pV030-8_ is depicted in green-blue. **(B)** Shows TraB_pIP501_ colored according to its orientation in the cell membrane. The grey part represents the secretion signal sequence; slate parts the extracellular part; the salmon parts are predicted to be within the membrane; the yellow parts are cytosolic. Amino acids, which are conserved in the multiple sequence alignments are shown in stick presentation. **(C)** and **(D)** Zoom into the areas of TraB_pIP501_ showing the highest conservation. The conserved amino acids are shown as sticks and labeled. Same color scheme as in panel **(B,C)** Showing the details of the interface area between the cell membrane and the peptidoglycan layer. **(D)** Zoom into the cytosolic loop of TraB_pIP501_, bottom view.

To obtain a more detailed picture of the 3D structure of the membrane-inserted TraB protein and its orthologs we generated structural models using the Robetta server (https://robetta.bakerlab.org). The recently available deep learning-based modeling method RoseTTAFold enables the ab-initio generation of structural models, which are quite accurate and do not require known homologous structures (Baek et al., 2021). The structures of TraB and the seven orthologs showed a very similar overall structure with the 2 TM helices arranged in an antiparallel manner and connected by a short and structurally defined loop. The signal helix, which is connected to TMH-1 *via* a longer, flexible loop exhibited the largest deviations in the alignment shown in Figure 3, panel A. The pairwise alignment to TraB_pIP501_ was performed with the alignment function using the cealign algorithm of *PyMOL 2.5* and yielded r.m.s.-deviations between 2.17 Å for the MPF protein from pUC11B over 80 atoms and 4.62 Å for TraB from *Staphylococcus aureus* 3688STDY6124879 over 104 atoms (Schrödinger and DeLano, 2020), *PyMOL* was retrieved from http://www.pymol.org/pymol. After removal of the secretion signal sequence the mature TraB protein consists of the two antiparallel TM helices, with a short flexible stretch at the N-terminal side of TMH-1 and the C-terminal end of TMH-2. The length of the two helices differs slightly (55 Å for TMH-1 and 50 Å for TMH-2) and matches quite well the secondary structure prediction and the expected length of a membrane spanning helix (Phillips, 2018). TMH-1 is straight, whereas TMH-2 features a pronounced kink in its C-terminal part, which is due to a conserved proline residue (P103 in TraB_pIP501_). The two TMHs are connected by a tight loop (P70 to P75), which constitutes one of the highest conserved regions in the multiple sequence alignment (Figure 3B, panel B).

The TM helices exhibit a distinct distribution of hydrophilic and hydrophobic residues: in the middle part (colored in salmon in Figure 3, panel B, C and D) there are mainly hydrophobic amino acids and no charged ones; the outer and inner parts (slate and yellow in Figure 3, panel B, C and D) consist of mainly hydrophilic residues, a large portion of which is charged. Both the outer and the inner parts carry a net positive charge, which is more pronounced for the inner part—including the TMH connecting loop, there are 5 lysines and 3 acidic residues (2 aspartates and 1 glutamate) in this region. The lysines are oriented closer to the central part of the TMHs consistent with their proposed membrane anchoring function (Landolt-Marticorena et al., 1993; Planque et al., 1999). The C-terminal part of TMH-2 has distinct features as it contains no charged amino acids but 3 aromatic residues, 2 tryptophanes and 1 tyrosine, which are clustered around the kink caused by P103. The two tryptophanes (W100 and W104 in TraB_pIP501_) are conserved among all compared TraB-like proteins and most likely play an important role in membrane anchoring (Landolt-Marticorena et al., 1993; Planque et al., 1999) and/or protein-protein interaction within the MPF complex. The completely conserved residues including the two tryptophanes are shown in Figure 3, panel B. For better visualization, we zoomed into the regions containing the conserved residues and labeled them referring to their position within TraB_pIP501_. This is shown in Figure 3, panel C for the outwards facing parts of TraB_pIP501_ and in panel D for the cytosolic loop region.

### *In Vitro* Cross-Linking Revealed TraB Oligomers in the Range of Dimers to Tetramers

**FIGURE 4:**
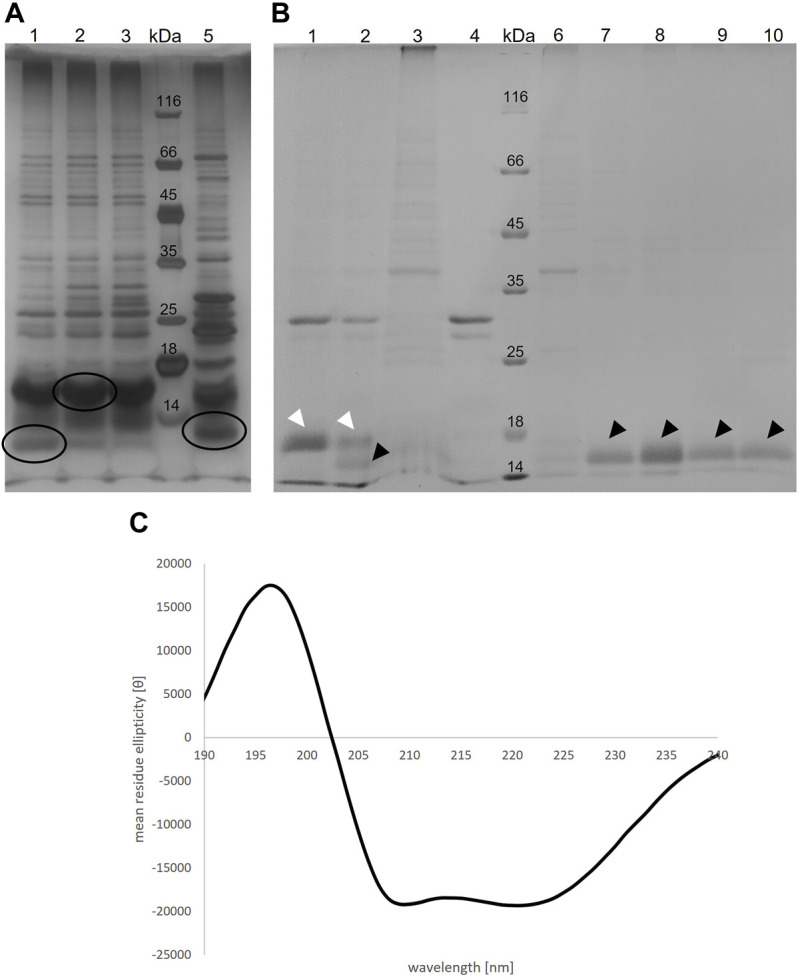
**(A)** Silver-stained 12% SDS polyacrylamide gel of the elution fractions from IMAC of the TraB_31-110_ test purification with two different *E. coli* expression strains. For lane 1-3 BL21 Codon Plus RIL cells were used, for lane 5 BL21 star cells. Lane 4 shows the MW standard (Thermo Fisher Scientific). Circled bands were cut out and applied to MS analysis **(B)** Coomassie-stained 12% SDS polyacrylamide gel showing different purification steps of TraB_31-110_. Lane 1: pooled elution fraction of the IMAC; lane 2: same fractions after TEV cleavage; lane 3 and 4: elution fractions of the reverse IMAC; lane 5: MW standard (Thermo Fisher Scientific); lane 6–10: elution fractions of the SEC using a Superdex 200 increase column. White arrow heads point at uncleaved TraB_31-110_. Black arrow heads point at TEV-cleaved TraB_31-110_. **(C)** CD spectrum of TraB_31-110_ showing two minima at 209 and 222 nm implying a mainly α-helical conformation of TraB_31-110_.

TraB_31-110_ encoded on pQTEV-*traB*_31-110_ was expressed in two different *E. coli* expression strains*,* BL21-CodonPlus (DE3)-RIL and BL21 star (DE3). A test purification, where the protein was isolated by a Ni-IMAC was performed to evaluate expression levels. As the isolated protein migrated slower than expected for its calculated MW, we performed MS analysis confirming that the band at 16 kDa corresponds to TraB_31-110_. The examined bands are shown in Figure 4, panel A. As the TraB_31-110_ band was more prominent using BL21-CodonPlus (DE3)-RIL cells for expression, they were used for large-scale expressions and purification. Figure 4, panel B shows the different purification steps of TraB_31-110_. Lanes 1 and 2 show the pooled elution fractions from the Ni-IMAC step before and after dialysis and TEV cleavage, respectively. TEV cleavage was performed for 2 days, because detergents might compromise the cleavage efficiency of the TEV protease. This appeared to be the case for the used Tween-20 as only about 50% of TraB_31-110_ was cleaved even after 2 days. Next, a reverse Ni-IMAC was performed to separate TEV-cleaved TraB_31-110_ from uncleaved TraB_31-110_ as well as from the TEV protease. Lanes 3 and 4 show the elution fractions of the reverse Ni-IMAC, containing impurities binding nonspecifically to the column, uncleaved TraB_31-110_ and TEV-protease. The flow through was collected, concentrated, and loaded on to a Superdex 200 increase column to obtain TraB_31-110_ of higher purity (lanes 6–10 of Figure 4, panel B). Lane 6 represents the peak eluting in the void volume of the column, containing highly aggregated impurities. TraB_31-110_ eluted as a single peak at an elution volume of 12.3 ml (lanes 7–10) corresponding to a calculated MW of 172 kDa. This represents the micelle bound TraB_31-110_. For further analysis like *in vitro* cross-linking or CD measurements only the main fractions containing TraB_31-110_ in the highest purity were used. The CD spectrum in Figure 4, panel C indicates mainly α-helical content and no β-strand which agrees with the structural features obtained from the secondary structure predictions of TraB_31-110_ (see *TraB is an α-Helical Transmembrane Protein*).

**FIGURE 5:**
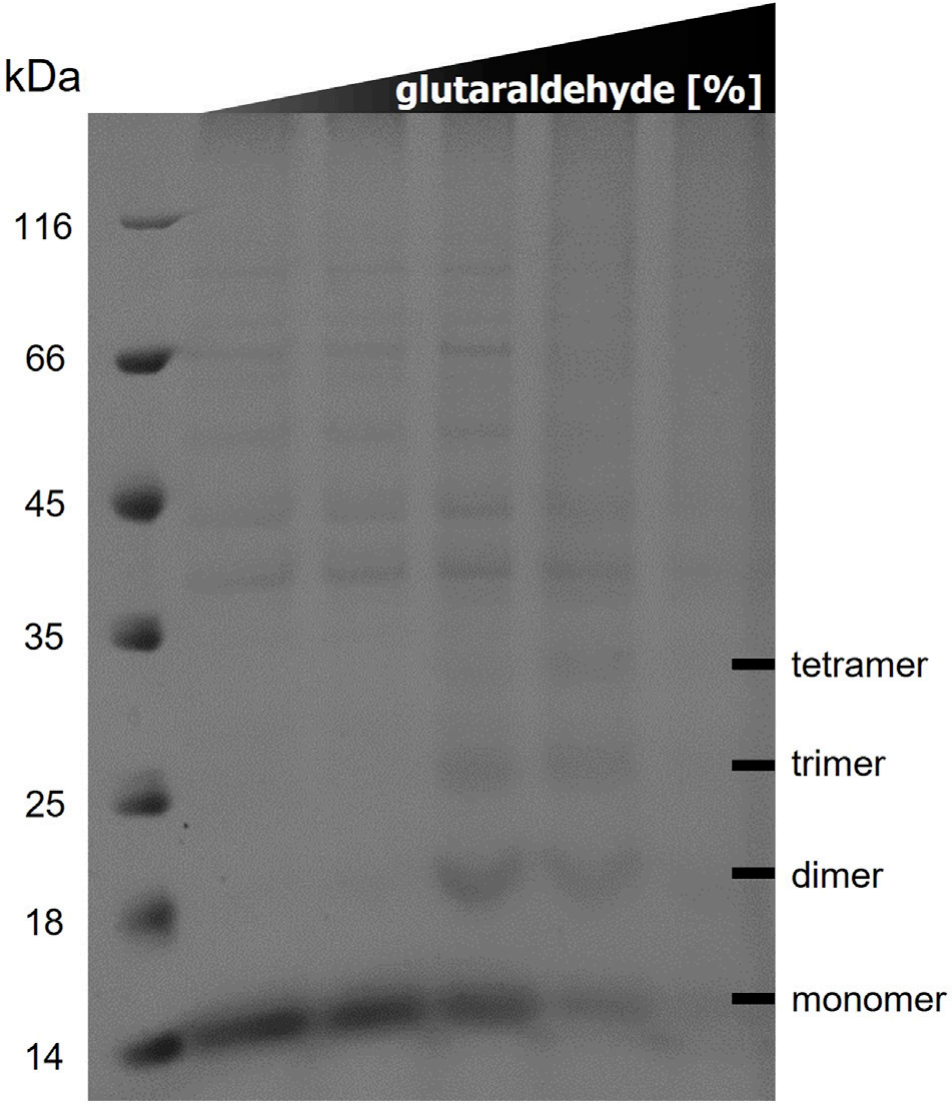
12% SDS polyacrylamide gel of *in vitro* cross-linking of TraB_31-110_ using increasing glutaraldehyde concentrations (0/0.001/0.01/0.05/0.1%) from left to right. Lane 1 shows the MW standard (Thermo Fisher Scientific).

The results of the *in vitro* cross-linking are shown in Figure 5. At a glutaraldehyde concentration of 0.01% a dimer and trimer band become clearly visible, and a faint band of a tetramer appears. At a crosslinker concentration of 0.05% the distribution is shifted towards the trimer and tetramer. At the highest glutaraldehyde concentration (0.1%) unspecific cross-linking occurred leading to an indistinguishable variety of aggregates of different sizes. These results indicate that TraB_31-110_ forms oligomers in Tween-20 micelles in a range from dimers to tetramers.

### TraB is Likely Part of the pIP501 MPF Complex

**FIGURE 6:**
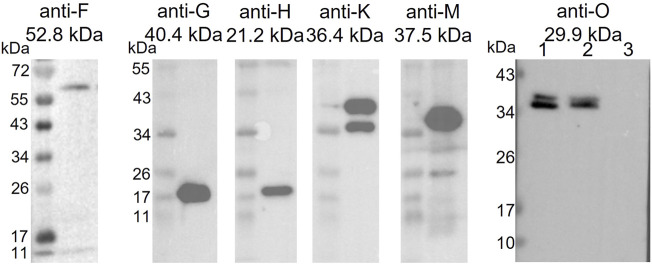
Western Blot of the elution fractions of the pull-down with TraB-Strep. The used anti-Tra antibody and the MW of the respective Tra_pIP501_ protein is given above the blot. The TraG protein runs significantly faster than its calculated MW. One possible explanation would be that only the CHAP domain of TraG is part of the eluted protein complex. The two TraK bands correspond to its two start codons resulting in proteins of sizes of 36.4 and 32.3 kDa. The Western Blot with anti-TraO antibody was performed as negative control. TraO is present in the lysate (lane 1) and in the flow through of the column (lane 2), but not in the elution fraction (lane 3). The two bands are showing TraO with and without its secretion sequence (the difference in MW = 2.5 kDa). The slower migration of the TraO bands may be explained by the high acidity of the proteins (pI 3.8 and 3.7) (Matagne et al., 1991).

As a predicted membrane protein, TraB is a likely candidate to interact with other T4SS proteins to assemble the translocation channel. A pull-down approach was used to elucidate the TraB interaction partners. The co-transformed *B. megaterium* MS941 strain expressed both, C-terminally Strep-tagged TraB (TraB-Strep) and the *tra*_pIP501_ operon proteins TraB to TraO. The C-terminal Strep-tag does not impact the functionality of TraB, as demonstrated by wild type transfer frequencies for the pIP501 *traB* knockout strain complemented with TraB containing a C-terminal Strep-tag (*E. faecalis* JH2-2 (pIP501∆*traB,* pEU327-RBS-*traB*-Strep) (Figure 1). TraB-Strep and interacting proteins were purified from the clarified supernatant of *B. megaterium* MS941 (pMGBM19-RBS-*traB*-*traO*; pRBBm59-RBS-*traB*-Strep) lysate *via* affinity chromatography. The putative MPF proteins TraF, TraH, TraM and TraK coeluted with TraB-Strep and were detected by immunoblotting (Figure 6). The cytosolic protein TraN and the putative surface adhesion protein TraO were used as negative controls. None of them was coeluted, as depicted for TraO (Figure 6). The two bands visible on the membrane incubated with anti-TraK antibody are due to two different translational start codons for TraK, resulting in the expression of two TraK variants differing by 4.1 kDa in size (Goessweiner-Mohr et al., 2014b). The two bands representing TraO are probably showing two differently processed forms. Unprocessed TraO has a MW of 29.9 kDa containing a putative secretion signal sequence with a cleavage site between alanine 24 and aspartic acid 25. Secreted TraO shows a MW of 27.4 kDa. The membrane incubated with anti-TraG antibody showed a band at around 20 kDa, which migrated significantly faster than the calculated MW of 40.4 kDa for TraG. This could mean that only the CHAP domain of TraG is part of the isolated protein complex as the band would correspond to its calculated MW of 22 kDa (Arends et al., 2013).

## Discussion

In this study, we focused on the small polytopic transmembrane protein TraB. We used biparental matings, pull-down assays and structural analyses to elucidate the potential functional role of TraB in the T4SS_pIP501_.

TraB was shown to be essential for conjugative pIP501 transfer. Biparental mating assays in *E. faecalis* revealed a total loss of transfer activity for the pIP501 *traB* knockout. The TraB N-terminal signal secretion sequence is crucial for transfer and location of TraB in the cell envelope. Cell fractionation of *E. faecalis* JH2-2 harboring pIP501, and *E. faecalis* JH2-2 pIP501∆*traB*, pEU327-RBS-*traB*-Strep showed its localization in the cell wall and cell membrane fraction (Figure 2 panel C) However, some residual transfer activity (mean transfer frequency of 1.53 × 10^−7^ transconjugants/recipient) was detected when only TraB_31-110_ was present. This residual transfer frequency could possibly result from an association of TraB with TraM or TraG, which could recruit TraB to the membrane *via* protein-protein interaction, as demonstrated in the pull-downs by co-elution of TraM and TraG. In addition, yeast two-hybrid studies suggested an interaction of TraB with TraG (Abajy et al., 2007).

Through pull-down assays, we confirmed the hypothesis that TraB is part of the MPF_pIP501_ complex. It could even act as one of the recruitment factors of the T4SS complex, likely together with the VirB8-like TraM protein and TraG, whose N-terminal TMH was shown to be required for correct membrane localization of TraM (Kohler et al., 2017). Due to inherent differences between the cell envelopes of Gram+ and Gram-bacteria, *B. megaterium* MS941 was used as an expression host. The strain does not express the major extracellular protease NprM in addition to its inherent alkaline protease deficiency (Wittchen and Meinhardt, 1995), making it a suitable host for the expression of native T4SS_pIP501_ proteins allowing a proper assembly of the MPF complex. The expression plasmid pMGBm19 harbors the pIP501 *tra* operon from *traB* to *traO* lacking the *traA* gene encoding the relaxase as conjugative relaxases are not involved in the MPF complex (Lang et al., 2010). TraA is essential for DNA transfer as it forms the single-stranded plasmid DNA to be transferred to the recipient, but the number of MPF complexes might be decreased in the presence of TraA as it controls gene expression of the *tra* operon by binding to *oriT* partially overlapping with the main *tra* operon promoter P_*tra*_.

TraF, TraH, TraK, TraM, and TraG were shown to coelute with TraB in the pull-downs. The VirB8 homologs TraM and TraH have been proposed as members of the T4SS core complex (Fercher et al., 2016; Kohler et al., 2017; Kohler et al., 2018b). TraF is a structural homolog of a T7SS component (A. Stallinger and W. Keller, personal communication) and together with TraC, TraI and VirB6-like TraL it is likely involved in T4SS complex formation (Abajy et al., 2007; Fercher et al., 2016). The cell envelope-located TraK is also a likely integral component of the core complex (Goessweiner-Mohr et al., 2014b). The lytic transglycosylase TraG was found to be crucial for the T4SS assembly but it is likely not present in the MPF complex itself as proposed in Arends et al*.* and Kohler et al*.* (Arends et al., 2013; Kohler et al., 2017). Interestingly, by probing the eluate of the TraB-Strep pull-down with the anti-TraG antibody, we found a protein whose size would fit the MW of the C-terminal domain of TraG, the CHAP domain. The appearance of a protein of the approximate size of the CHAP domain in the eluate is intriguing. We speculate that a hitherto uncharacterized intracellular peptidase in *B. megaterium* might have cleaved the flexible linker region between the SLT and CHAP domain in TraG (Arends et al., 2013; Jeong et al., 2018). Thus, we cannot exclude TraG being a member of the T4SS complex, although the interaction of TraG with the members of the T4SS complex could be only transient.

In addition, in a pull-down performed with TraK-Strep, the same proteins were coeluted, as here demonstrated for the TraB-Strep based pull-down, namely TraF, TraG, TraH, and TraM (data not shown). These data are consistent with the results presented in this study.

For all orthologs of TraB_pIP501_ a similar domain structure and topology was predicted. All orthologs feature a secretion signal sequence, which is cleaved upon secretion, resulting in a mature two-helix transmembrane protein. The most conserved regions were parts of the TMH and the short cytoplasmic loop connecting TMH-1 and TMH-2 (Ditty and Harwood, 1999).

In addition, we created a phylogenetic tree using MEGA version 11 (Tamura et al., 2021) using a higher number of orthologs, including several CagC family proteins. As CagC was proposed to be the major pilin of the *Helicobacter pylori* T4SS, we included two prominent pilin proteins, VirB2 of *Agrobacterium tumefaciens* and *Brucella suis*. The results are shown in Supplementary Figure S1 and suggest that there is no relevant relation between TraB_pIP501_ and the two pilins. Nevertheless, we aligned their theoretical structure with the theoretical structure of TraB_pIP501_ and show this superposition in Supplementary Figure S2. In spite of the topological similarity of their mature forms (two antiparallel α-helices connected by a short loop) the superposition shows a pronounced structural difference and results in a high RMSD deviation. A prominent feature, the kink in the C-terminal α-helix, is common to all presented orthologs, but is missing in the theoretical pilin structures.

The alignment of the theoretical 3D structures of the TraB orthologs in Figure 3 shows a high structural conservation for TMH-1 and TMH-2 among all orthologs. The highest differences were found in the orientation and length of the first helix, and in the conformation of the flexible linker, which is also part of the secretion signal sequence.

The conserved features of all TraB orthologs such as the length and anti-parallel arrangement of the TMHs, the kink in TMH-2, the tight loop between TMH-1 and TMH-2 and the structurally conserved position of charged and aromatic residues suggest that TraB_pIP501_ and its orthologs play an important role in the formation of the MPF complex. As TraB_pIP501_ is the only protein encoded by the pIP501 *tra* operon (except for TraO that is not part of the MPF), which features a secretion signal sequence, one role of TraB could be that of a recruitment factor for some of the other Tra proteins, which may not be able to self-insert into the membrane. The drastically reduced transfer rate when using TraB_31-110_ instead of wild type TraB also corroborates its role as a putative recruitment factor. Based on the length and arrangement of the TMHs TraB is also likely to adopt a structural role in building up the MPF complex: the length is consistent with the proposed thickness of the bacterial membrane and the conserved positively charged and aromatic residues are ideally positioned for anchoring the protein in the membrane. As we showed in the *in vitro* cross-link, TraB can form defined oligomers in detergent micelles. The highest oligomerization was found to be a tetramer; however, this might be due to the restriction in size of the protein-micelle complex. In the context of the MPF complex it is well conceivable that TraB forms higher oligomers by self-interaction and possible interactions with other members of the pIP501 T4SS.

Interestingly, TraB orthologs were found on a variety of bacterial species belonging to the phylum Firmicutes. They include putative T4SSs from environmental strains, such as *C. algoriphilum* which was first isolated from water brine in permafrost (Shcherbakova et al., 2005) as well as conjugative plasmids from severe nosocomial pathogens such as *S. aureus*, MRSA and *E. faecalis*. pRE25 and pAMβ1 are, like pIP501, broad-host-range multiresistance plasmids of the inc18 incompatibility group which can transfer virtually to all Gram + bacteria (Kohler et al., 2018b). pV030-8, which was isolated from *S. aureus*, belongs to the pSK41/pGO1 family of multiresistance plasmids capable of promoting conjugative resistance transfer among staphylococci (Firth and Skurray, 2006; Pérez-Roth et al., 2010). The presence of TraB orthologs on T4SSs from a variety of bacterial genera including environmental, biotechnologically relevant as well as pathogenic species points to a crucial role of this small transmembrane protein in the horizontal dissemination of diverse factors including antibiotic resistance genes.

## Data Availability

The original contributions presented in the study are included in the article/Supplementary Material, further inquiries can be directed to the corresponding authors.
