# Supplementary figures and images for: Small Things Matter: The 11.6-kDa TraB Protein is Crucial for Antibiotic Resistance Transfer Among Enterococci
Source: Front Mol Biosci. 2022 Apr 25;9:867136. doi: 10.3389/fmolb.2022.867136 (PMC9083827; doi:10.3389/fmolb.2022.867136)

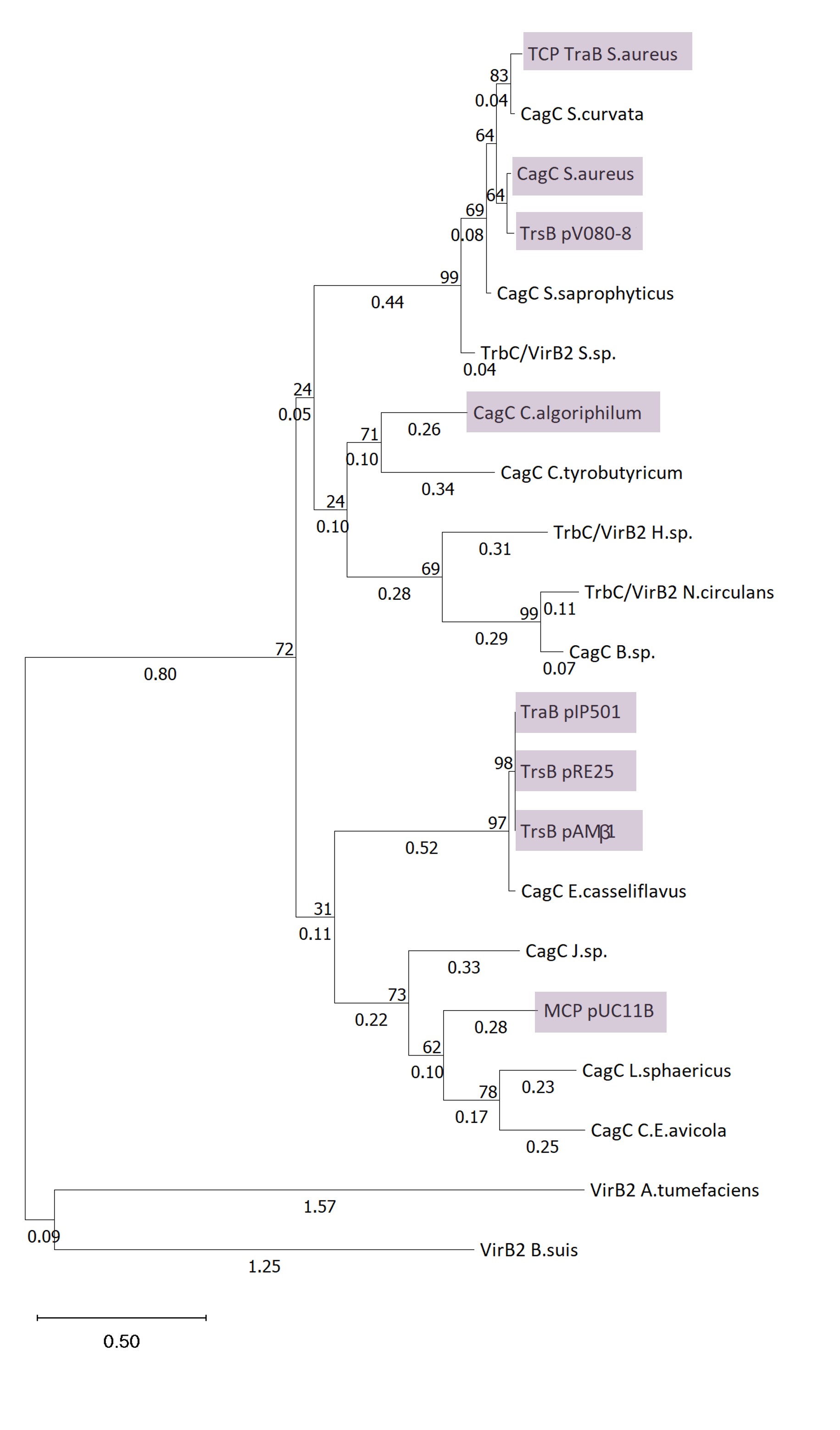

Supplement: Supplementary file 1 [file Image1.JPEG]

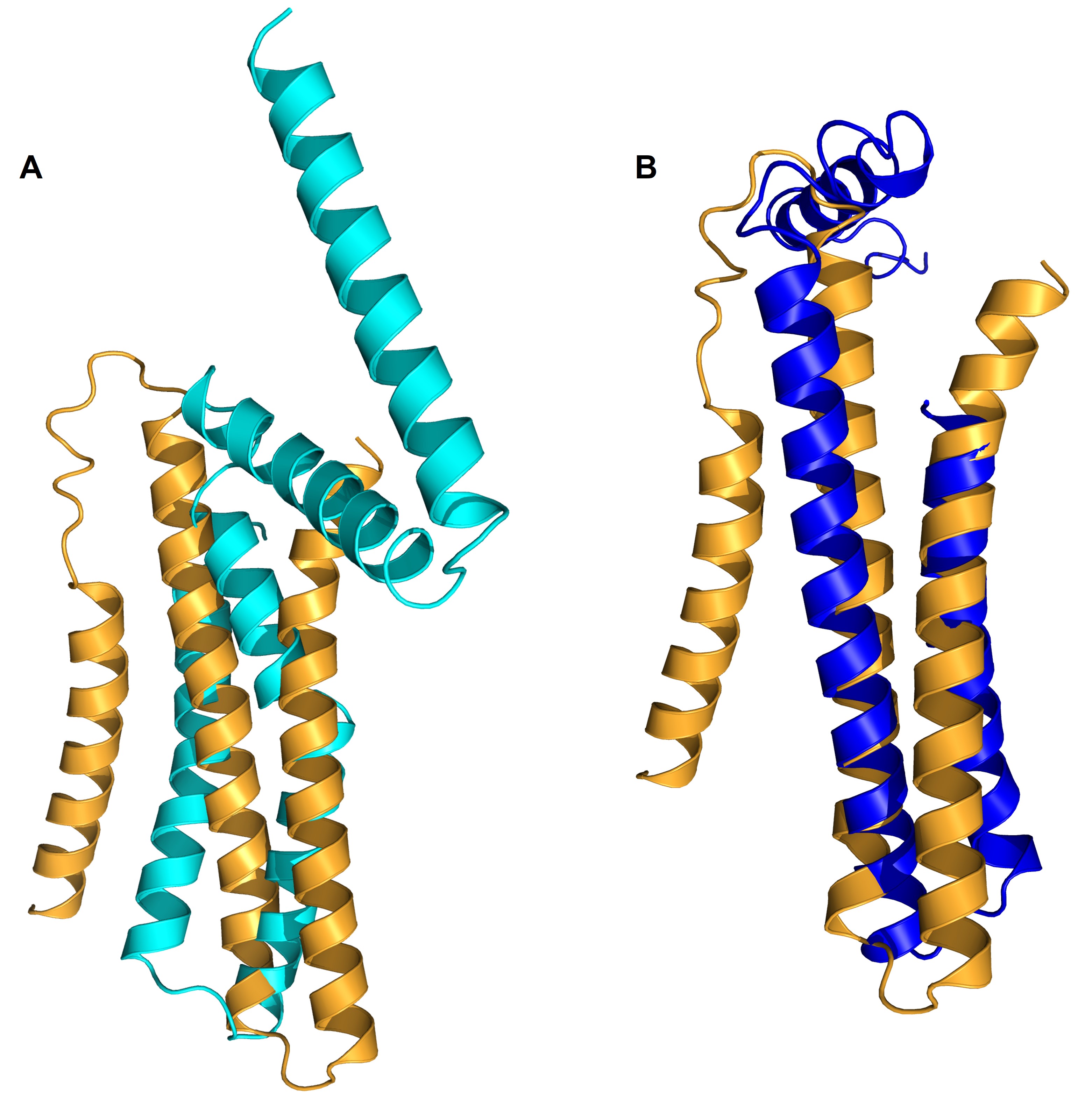

Supplement: Supplementary file 2 [file Image2.JPEG]
