# Supplementary material for: Small Things Matter: The 11.6-kDa TraB Protein is Crucial for Antibiotic Resistance Transfer Among Enterococci
Source: Front Mol Biosci. 2022 Apr 25;9:867136. doi: 10.3389/fmolb.2022.867136 (PMC9083827; doi:10.3389/fmolb.2022.867136)
Supplement: Supplementary file 3 [file DataSheet1.docx]

Supplementary Material

# Supplementary Data

Supplementary Table 1 Bacterial strains and plasmids used in this work

| **Strain or plasmid** | | | **Genotype or description** | **Selection** | **Reference or source** |
| --- | --- | --- | --- | --- | --- |
|  | | |  |  |  |
| **Strains** | | |  |  |  |
|  | *Bacillus megaterium* | |  |  |  |
|  |  | MS941 | ∆*nprM* | - | Mobitec GmbH, Göttingen, Germany |
|  | *Escherichia coli* | |  |  |  |
|  |  | BL21-CodonPlus (DE3)-RIL | F^–^ *ompT hsdS* (r_B_ ^–^ m_B_ ^–^) *dcm*^+^ Tet^R^ *gal* λ(DE3) *endA* Hte [*argU ileY leuW* Cm^r^] | Cm (35 µg/ml) | Stratagene, San Diego, California, USA |
|  |  | BL21 Star™ (DE3) | F^_^ *ompT hsdSB* (r_B_^-^ m_B_^-^) *gal* *dcm* *rne131* | - | Thermo Fisher Scientific, Waltham Massachusetts, USA |
|  |  | DH5α | *fhuA2* Δ*(argF-lacZ) U169 phoA glnV44* Φ*80*Δ *(lacZ)M15 gyrA96 recA1 relA1 endA1 thi-1 hsdR17* | - | New England Biolabs, Frankfurt am Main, Germany |
|  |  | EC1000 | F^-^ RepA^+^ *araD139* (*araABC-leu*)7679 *galU galK lacX74 rspL thi;* Kan^R^ | Kan (50 µg/ml) | Leenhouts et al., 1996 |
|  |  | TOP10 | F^-^ *mcrA* ∆(*mrr-hsd*RMS-*mcr*BC) Φ80*lacZ*∆M15 ∆*lac*X74 *rec*A1 *ara*D139 ∆(*ara-leu*) 7697 *gal*U *gal*K *rps*L (Str^R^) *end*A1 *nup*G λ- | - | Thermo Fisher Scientific, Waltham Massachusetts, USA |
|  | *Enterococcus faecalis* | |  |  |  |
|  |  | JH2-2 | Derivative of *E. faecalis* JH2, Rif^R^, Fus^R^ | Fus (50 µg/ml) | Jacob and Hobbs, 1974 |
|  |  | OG1X | Protease-negative mutant of *E. faecalis* OG1-10, Sm^R^ | Sm (1.5 mg/ml) | Ike et al., 1983 |
| **Plasmids** | | |  |  |  |
|  | pEU327 | | *E. coli/*G+ bacteria shuttle plasmid, Spec^R^, *xylA* promoter | Spec (100 µg/ml)^*^  Spec (500 µg/ml)^**^ | Eichenbaum et al., 1998 |
|  | pEU327-RBS-*traB* | | pEU327 with RBS-*traB*, Spec^R^ | Spec (100 µg/ml)^*^  Spec (500 µg/ml)^**^ | This study |
|  | pEU327-RBS-*traB*_31-110_ | | pEU327 with RBS-*traB*_31-110_, Spec^R^ | Spec (100 µg/ml)^*^  Spec (500 µg/ml)^**^ | This study |
|  | pEU327-Strep | | pEU327 with C-terminal Strep-tag II (SA-WSHPQFEK) and 3’ stop codon, Spec^R^ | Spec (100 µg/ml)^*^  Spec (500 µg/ml)^**^ | This study |
|  | pEU327-RBS-*traB*-Strep | | pEU327 with RBS-*traB*, C-terminal Strep-tag II and 3’ stop codon, Spec^R^ | Spec (100 µg/ml)^*^  Spec (500 µg/ml)^**^ | This study |
|  | pMGBm19 | | *B. megaterium*/*E. coli* shuttle vector, Cm^R^, Amp^R^, xylose-inducible promoter, *xyl* repressor, pBM100 replicon, ColE1 origin | Cm (35 µg/ml) | Mobitec GmbH, Göttingen, Germany |
|  | pMGBm19-RBS-*traB*-*traO* | | pMGBm19 with *traB-traO*_pIP501_ inserted at BamHI and SacI sites with additional 5’ RBS, Cm^R^, Amp^R^ | Cm (35 µg/ml) | This study |
|  | pIP501 | | *tra*^+^, Cm^R^, MLS^R^ | Cm (20 µg/ml) | Evans and Macrina, 1983 |
|  | pIP501∆*traB* | | pIP501 *traB* in-frame deletion, Cm^R^, MLS^R^ | Cm (20 µg/ml) | This study |
|  | pKA | | pORI280 derivative, *oriT*_pCF10_, P‑*pheS*,* pCJK47 *aacA-aphD* at BglII site, Em^R^, Gent^R^ | Em (250 µg/ml) | Arends et al., 2013 |
|  | pKA-UPS-DWS-*traB* | | pKA with *traB* up- and downstream regions at PstI/EcoRI sites, Em^R^, Gent^R^ | Em (250 µg/ml) | This study |
|  | pQTEV | | P_t4_ *lacI*^q^ His_7_ Amp^R^ | Amp (100 µg/ml) | Scheich et al., 2004 |
|  | pQTEV-*traB*_31-110_ | | pQTEV with *traB*_31-110_, Amp^R^ | Amp (100 µg/ml) | This study |
|  | pRBBm59 | | *B. megaterium*/*E. coli* shuttle vector, Tet^R^, Amp^R^, sucrose-inducible promoter P*_sacB_*, *repU* replicon, pBR322 origin | Tet (10 µg/ml)^***^  Amp (100 µg/ml)^*^ | Addgene, Cambridge, Massachusetts, USA |
|  | pRBBm59-RBS-*traB*-Strep | | pRBBm59 with C-terminally Strep-tagged *traB*_pIP501_ and artificial 5’ RBS, Tet^R^, Amp^R^ | Tet (10 µg/ml)^***^  Amp (100 µg/ml)^*^ | This study |
|  | pUC18 | | Amp^R^ | Amp (100 µg/ml) | Promega, Walldorf, Germany |
|  | pUC18-UPS-*traB* | | pUC18 with *traB* upstream region at PstI/XbaI sites, Amp^R^ | Amp (100 µg/ml) | This study |
|  | pUC18-UPS-DWS-*traB* | | pUC18-UPS-*traB* with *traB* downstream region at BamHI/EcoRI sites, Amp^R^ | Amp (100 µg/ml) | This study |

Amp^R^, ampicillin resistance; Cm^R^, chloramphenicol resistance; Em^R^, erythromycin resistance; Fus^R^, fusidic acid resistance; Gent^R^, gentamicin resistance; Kan^R^, kanamycin resistance; MLS^R^, macrolide-lincosamide-streptogramin B resistance, Rif^R^, rifampicin resistance; Spec^R^, spectinomycin resistance; Sm^R^, streptomycin resistance; Tet^R^, tetracycline resistance; RBS, ribosomal binding site; *tra*^+^, transfer proficient; antibiotic concentration used for selection in ^*^ *E. coli*, ^**^ *E. faecalis* and ^***^ *B. megaterium* given in brackets.

Supplementary Table 2 Oligonucleotides used in this work

| **Name** | **Sequence (5‘-3‘)** | **Nucleotide position/reference** |
| --- | --- | --- |
| ***traB*_31-110_ expression cloning** | |  |
| BamHI_traB_31-110_ fw | TATA**GGATCC**GCCACTGATCCACAAG | 3493-3508 |
| HindIII_traB_31-110_ rev | TATA**AAGCTT**TTAAGCAAGTAAGCCATACGCC | 3714-3735 |
| ***traB* in-frame deletion** | |  |
| PstI_ups for orf2 ko fwd | GCG**CTGCAG**AAACAACGTTTGATGTTAAGT | 2402-2422 |
| XbaI_ups for orf2 ko rev | GGC**TCTAGA**TTTCATTTTATACACCTCTTG | 3388-3408 |
| BamHI_dws for orf2 ko fwd | CAG**GGATCC**GCTTAATAGAGAAGGGAG | 3730-3747 |
| EcoRI_dws for orf2 ko rev | CGC**GAATTC**CTAACTATTCAAAACCTTTTT | 4763-4785 |
| **pKA sequencing** | |  |
| pKA fw | GGAGACTACTTATTATGTAA | (Kohler et al., 2018) |
| pKA rev | GCGCTTGTAATGTCATAT | (Kohler et al., 2018) |
| **Screening in-frame deletion** | |  |
| A Test delta traB fw | ACAAGACCAAAACTTTACTT | 2221-2240 |
| B Test delta traB rev | ACGTGGCTTATTGTTGTAT | 4940-4958 |
| C Test delta traB fw | AACAAGAGGTGTATAAAATG | 3386-3405 |
| D Test delta traB rev | TTCATAACTAACTCCCTTC | 3740-3758 |
| ***traB* complementation** | |  |
| pEU327_SalI_RBS traB rev | GGC**GTCGAC**CCTTCTCTATTAAGCAAGT | 3726-3745 |
| pEU327_BstYI_RBS traB fw | CGC**GGATCT**TAAAAACAAGAGGTGTATAAA | 3382-3402 |
| Mut_pEUtraB31-110 fw | CATTTTATACACCTCTTGTTTTTAGGATC | 3382-3405 |
| Mut_pEUtraB31-110 rev | GCCACTGATCCACAAGCG | 3493-3510 |
| pEU327_Mut_C-Strep fw | GGTGGCTCCAAGCGCTATCGATCCGGCATTCAAATAC | This study |
| pEU327_Mut_C-Strep rev | CGCAGTTCGAGAAATAAAGATCCTCTAGAGTCGAC | This study |
| GA_RBS-traB fw | GTGGCTCCAAGCGCTAGCAAGTAAGCCATACGC | 3715-3732 |
| GA_RBS-traB rev | TGAATGCCGGATCGATTAAAAACAAGAGGTGTATAAAATGAAAAAATTTATC | 3382-3417 |
| GA_pEU327_C Strep fw | ATCGATCCGGCATTCAAATACAGATGCATTTTATTTC | This study |
| GA_pEU327_C Strep rev | AGCGCTTGGAGCCACCCG | This study |
| **pEU327 sequencing** | |  |
| pEU327 fw | CTTGCCAGTCACGTTACG | (Eichenbaum et al., 1998) |
| pEU327 rev | GATCAGCGATATCCACTTC | (Eichenbaum et al., 1998) |
| **pMGBm19 cloning** | |  |
| pMGBm19-traBO_fw | ATAT**GAGCTC**AATAAAAAGGGGGAAACAACAAATGAAAAAATTTATCAAAGATACAAAG | This study |
| pMGBm19-traBO_rev | ATATAT**GGATCC**TTAGATTCTCTTTTTAAAAATGAATGCTGA | This study |
| **pRBBm59 cloning** | |  |
| traB-pRBBm59 fw | GACTAAAAAGGGGGAAA**GGATCC**ATGAAAAAATTTATCAAA | This study |
| traB-pRBBm59 rev | ATATTATTTCTCGAACTGCG | This study |
| GA_pRBBm59 fw | TGAATTCGCGGCCGCATG | This study |
| GA_pRBBm59 rev | CCAGATCTTCGAACTAGTAGCAGC | This study |
| GA_traB CStrep prBB fw | CTAGTTCGAAGATCTGGTAAAAAGGGGGAAAGGAT | This study |
| GA_traB CStrep prBB rev | GCGGCCGCGAATTCATTATTTCTCGAACTGCG | This study |
| **pRBBm59 sequencing** | |  |
| Screen_pRBBm59 fw | CGCAACGTCTGGAAATCGTG | This study |
| Screen_pRBBm59 rev | GCGCATTCACAGTTCTCCGC | This study |
| restriction sites are shown in bold, ribosomal binding sites are underlined  GenBank accession number for pIP501 sequences used in this study is L39769.1  All primers without reference in the table were designed in this study. | |  |


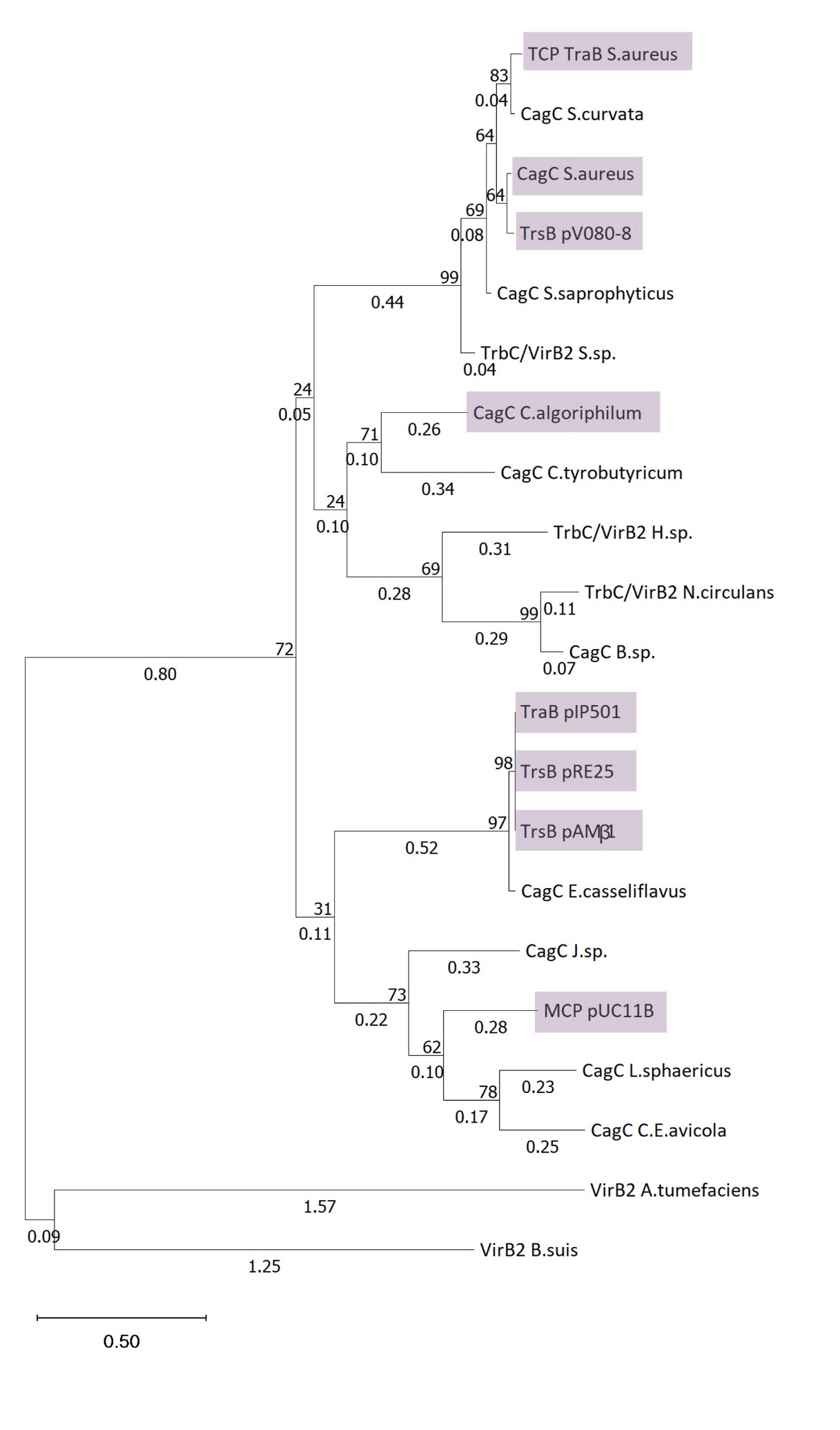


Supplementary Figure 1: Phylogenetic Tree created using MEGA version 11 (Tamura et al., 2021) based on PSI-BLAST using the NCBI database. For the required multiple sequence alignment two known pilin proteins were added manually as VirB2 like proteins were found in the search for orthologs. TCP: transfer channel protein; CagC: CagC family type IV secretion system protein; MCP: mating channel formation protein. If encountered on a plasmid, the plasmid name is given. If location of the gene (genome or plasmid) is not specified, the bacterial strain is given. [S. aureus]: [*Staphylococcus aureus*], [C. algoriphilum]: [*Clostridium algoriphilum*], [E. casseliflavus]: [*Enterococcus casseliflavus*], [L. sphaericus]: [*Lysinibacillus sphaericus*], [J. sp.]: [*Jeotgalibaca* species], [C. E. avicola]: [*Candidatus Enterococcus avicola*], [N. circulans]: [*Niallia circulans*], [S. saprophyticus]: [*Staphylococcus saprophyticus*],[C. tyrobutyricum]: [*Clostridium tyrobutyricum*], [S. sp.]: [*Staphylococcus species*], [B. sp.]: [*Bacillus species*], [H. sp]: [*Halolactibacillus species*], [S. curvata]: [*Shinella curvata*], [B. suis]: [*Brucella suis*], [A. tumefaciens]: [*Agrobacterium tumefaciens*]. Distance values are shown below the branches. Values lower than 0.04 are not shown. Bootstrap values are depicted at branch nodes. Orthologs used for detailed analysis are highlighted with a lavender background.


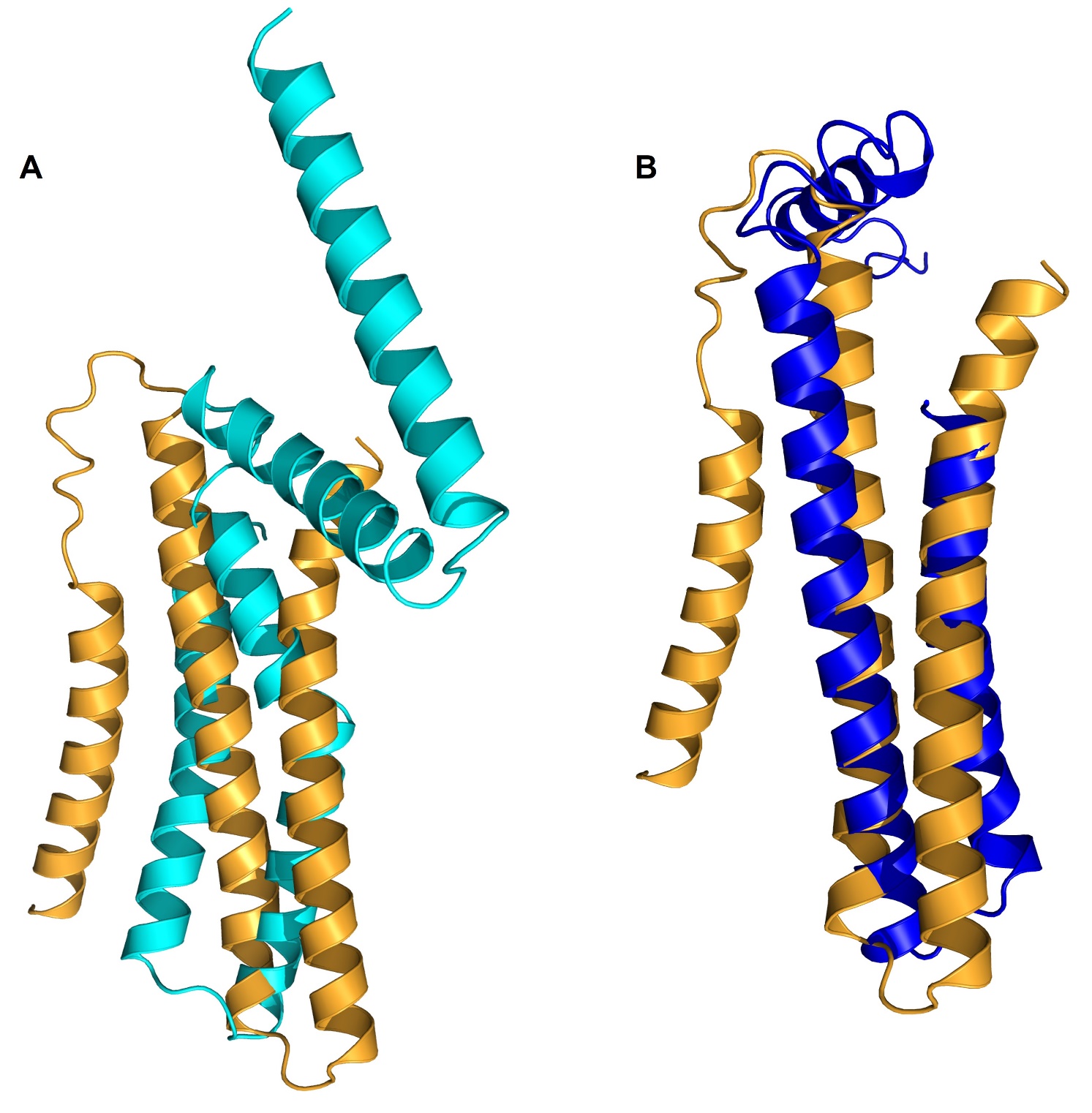


Supplementary Figure 2: Alignment of the theoretical structure of TraB_pIP501_ (shown in orange) with two typical pilin proteins. (A) shows the structural alignment with VirB2 from *Agrobacterium tumefaciens* 15955 depicted in turquoise. (B) shows the alignment with VirB2 from *Brucella suis* biovar 1 1330 depicted in blue. Structures were generated using RoseTTAFold.

REFERENCES

Arends, K., Celik, E.-K., Probst, I., Goessweiner-Mohr, N., Fercher, C., Grumet, L., et al. (2013). TraG encoded by the pIP501 type IV secretion system is a two-domain peptidoglycan-degrading enzyme essential for conjugative transfer. *J Bacteriol* 195, 4436–4444. doi: 10.1128/JB.02263-12

Eichenbaum, Z., Federle, M. J., Marra, D., Vos, W. M. de, Kuipers, O. P., Kleerebezem, M., et al. (1998). Use of the lactococcal *nisA* promoter to regulate gene expression in gram-positive bacteria: comparison of induction level and promoter strength. *Appl Environ Microbiol* 64, 2763–2769. doi: 10.1128/AEM.64.8.2763-2769.1998

Evans, R. P., and Macrina, F. L. (1983). *Streptococcal* R plasmid pIP501: endonuclease site map, resistance determinant location, and construction of novel derivatives. *J Bacteriol* 154, 1347–1355. doi: 10.1128/jb.154.3.1347-1355.1983

Ike, Y., Craig, R. A., White, B. A., Yagi, Y., and Clewell, D. B. (1983). Modification of *Streptococcus faecalis* sex pheromones after acquisition of plasmid DNA. *Proc Natl Acad Sci U S A* 80, 5369–5373. doi: 10.1073/pnas.80.17.5369

Jacob, A. E., and Hobbs, S. J. (1974). Conjugal transfer of plasmid-borne multiple antibiotic resistance in *Streptococcus faecalis* var. *zymogenes*. *J Bacteriol* 117, 360–372. doi: 10.1128/jb.117.2.360-372.1974

Kohler, V., Goessweiner-Mohr, N., Aufschnaiter, A., Fercher, C., Probst, I., Pavkov-Keller, T., et al. (2018). TraN: A novel repressor of an *Enterococcus* conjugative type IV secretion system. *Nucleic Acids Res* 46, 9201–9219. doi: 10.1093/nar/gky671

Leenhouts, K., Buist, G., Bolhuis, A., Berge, A. ten, Kiel, J., Mierau, I., et al. (1996). A general system for generating unlabelled gene replacements in bacterial chromosomes. *Mol Gen Genet* 253, 217–224. doi: 10.1007/s004380050315

Scheich, C., Niesen, F. H., Seckler, R., and Büssow, K. (2004). An automated *in vitro* protein folding screen applied to a human dynactin subunit. *Protein Sci* 13, 370–380. doi: 10.1110/ps.03304604

Tamura, K., Stecher, G., and Kumar, S. (2021). MEGA11: Molecular Evolutionary Genetics Analysis Version 11. *Mol Biol Evol* 38, 3022–3027. doi: 10.1093/molbev/msab120
